# High-Sensitivity Troponins and Homocysteine: Combined Biomarkers for Better Prediction of Cardiovascular Events

**DOI:** 10.3390/ijms26178186

**Published:** 2025-08-23

**Authors:** Dragan Djuric, Zorislava Bajic, Nina Radisavljevic, Tanja Sobot, Slavica Mutavdzin Krneta, Sanja Stankovic, Ranko Skrbic

**Affiliations:** 1 Institute of Medical Physiology “Richard Burian”, Faculty of Medicine, University of Belgrade, 11000 Belgrade, Serbia; nina_radisavljevic@outlook.com (N.R.); slavica.mutavdzin@med.bg.ac.rs (S.M.K.); 2 Centre for Biomedical Research, Faculty of Medicine, University of Banja Luka, 78 000 Banja Luka, Bosnia and Herzegovina; zorislava.bajic@med.unibl.org (Z.B.); tanja.sobot@med.unibl.org (T.S.); ranko.skrbic@med.unibl.org (R.S.); 3 Department of Physiology, Faculty of Medicine, University of Banja Luka, 78 000 Banja Luka, Bosnia and Herzegovina; 4 Centre of Medical Biochemistry, University Clinical Centre of Serbia, 11000 Belgrade, Serbia; sanjast2013@gmail.com; 5 Faculty of Medical Sciences, University of Kragujevac, 34000 Kragujevac, Serbia; 6 Department of Pharmacology, Toxicology and Clinical Pharmacology, Faculty of Medicine, University of Banja Luka, 78 000 Banja Luka, Bosnia and Herzegovina; 7 Department of Pathologic Physiology, I.M. Sechenov First Moscow State Medical University, 119435 Moscow, Russia

**Keywords:** cardiovascular disease, high-sensitivity cardiac troponins, homocysteine, cardiovascular risk stratification

## Abstract

As the leading cause of global mortality, cardiovascular diseases demand improved and innovative strategies for early detection and risk assessment to enhance prevention and timely treatment. This comprehensive review examines the potential of combining high-sensitivity cardiac troponins (hs-cTns) and homocysteine (Hcy) as complementary biomarkers for enhanced cardiovascular risk prediction. hs-cTn assays have revolutionized cardiovascular diagnostics by enabling the detection of minimal myocardial injury, improving early diagnosis of acute coronary syndrome, and providing robust prognostic information in both symptomatic and asymptomatic populations. Hcy, while established as a marker of vascular dysfunction, presents an interpretative challenge due to multiple confounding factors and inconsistent therapeutic responses. Emerging evidence demonstrates significant correlations between elevated Hcy and troponins across various clinical conditions, suggesting that their combined assessment—reflecting both myocardial injury and vascular dysfunction—may improve cardiovascular risk stratification. While initial findings are promising, additional studies are required to validate the clinical value of the combined marker approach. Future development of personalized interpretation algorithms, and multi-marker panels incorporating these biomarkers, may significantly advance cardiovascular medicine and enable more effective population-specific risk management strategies.

## 1. Introduction

In the last half-century, significant efforts to find a suitable biochemical marker for cardiovascular disease (CVD) have led to the identification of multiple markers and the refining of detection methodologies [1].

The identification of serum aspartate aminotransferase (AST), which was formerly called glutamic oxaloacetic transaminase (SGOT), occurred in 1954 [2]. A technique for assessing AST was established by Karmen [3] and subsequently refined and enhanced by Henry and colleagues [3,4]. Later on, Hill and Levi detected lactate dehydrogenase (LDH) in the blood samples [5], while Wróblewski and LaDue found an increase in the activity of this enzyme among patients with myocardial injury [2,6]. Efforts to create a precise and sensitive approach for measuring creatine kinase (CK) activity have been ongoing since 1955. It took the medical community nearly ten years to acknowledge its importance as a potential symptom of heart-related disorders [1,7]. In 1972, a pivotal contribution was made by Roe and his team through the development of a technique to assess the CK-MB isoenzyme [8], which subsequently facilitated its integration into diagnostic applications for acute myocardial infarction (MI), as established by the World Health Organization (WHO) [9]. In 1978, myoglobin was suggested as a potential indicator for CVD. However, its limited use stemmed from its lack of specificity and the quick clearance from the circulatory system [1].

In the early 1960s, Ebashi revealed that calcium ions are fundamental to the molecular mechanisms of skeletal muscle contraction, stressing their importance in the troponin– tropomyosin complex [10,11]. The revelation from 1971 indicated that the troponin complex is made up of three parts: troponin I (TnI), which functions as an ATPase inhibitor; troponin C (TnC), which exhibits a strong affinity for Ca^2+^; and troponin T (TnT), which binds to tropomyosin [12]. The genes encoding the troponin (Tn) subunits have evolved separately, producing isoforms characteristic of various muscle fiber types. TnC exists in two isoforms: one in fast skeletal muscle and the other in slow and cardiac muscles. Conversely, TnI and TnT have evolved from a TnI-like progenitor, exhibiting three isoforms specific to slow, fast, and cardiac muscles [13,14]. Skeletal muscle cells express specific Tn isoforms, like slow TnI and TnT, in pure slow fibers, and fast TnI and TnT in pure fast fibers [15,16].

C-reactive protein (CRP) is a key inflammatory biomarker for assessing cardiovascular risk, with high-sensitivity CRP (hs-CRP) recognized as a risk enhancer by American guidelines. However, its role in risk stratification is debated [17]. Galectin 3 (Gal-3) and the soluble ST2 receptor (sST2r) are two new biomarkers related to inflammation and fibrosis in heart failure (HF) [18,19]. Gal-3 is crucial in inflammatory responses and tissue fibrogenesis, promoting collagen production and cardiac remodeling after damage. However, it is not specific to heart conditions as its levels can rise in infections or chronic kidney disease. sST2r may be a prognostic biomarker for HF, unaffected by kidney function, but cannot be used for diagnosis due to potential elevation in various inflammatory conditions [20]. Natriuretic peptides are dependable biomarkers for HF and possess significant prognostic value. The atrial natriuretic peptide (ANP) and B-type natriuretic peptide (BNP) are the only cardiac natriuretic peptides currently known. These markers are released because of myocardial stretching. Pharmaceuticals can affect circulating natriuretic peptide levels by altering their secretion or clearance. Many medications, like SGLT-2 inhibitors, diuretics, renin–aldosterone–angiotensin system blockers, and others, may decrease natriuretic peptides, while beta-blockers, digitalis, and aspirin increase their levels [21].

At the turn of the 20th and 21st centuries, scientists became increasingly interested in identifying new risk factors for CVDs, such as serum homocysteine (Hcy) levels. In 1932, Hcy was isolated from bladder stones by Butz LW and Du Vigneaud V, who received the Nobel Prize in Chemistry in 1955 [22]. It is a cytotoxic sulfur-containing, nonessential amino acid with S-methyl groups, and serves as an essential intermediate in both the methionine cycle and cysteine metabolism. Elevated Hcy levels, known as hyperhomocysteinemia (HHcy), are recognized as a separate risk factor for CVDs [23,24]. In 1969, Kilmer McCully [25], a pathologist at Harvard, reported on two children with homocystinuria, a genetic disorder marked by the presence of Hcy in the urine due to severe HHcy levels exceeding 100 μmol/L. Both children exhibited vascular problems. One case involved a 2-month-old boy who showed advanced arteriosclerosis, resembling that typically seen in elderly individuals with severe CVD. The other child, an 8-year-old, tragically died from a stroke related to arteriosclerosis in the carotid artery. These cases were among the first indications that elevated plasma Hcy might be a possible cause of premature vascular disease, highlighting smooth muscle proliferation, arterial stenosis, and hemodynamic changes. Following 1975, Dr. McCully established a theoretical framework for the hypothesis that Hcy contributes to atherosclerosis. In 1976, his theory received validation from Wilcken DEL and Wilcken B, who conducted and published the first clinical study demonstrating the association of Hcy with coronary heart disease (CHD). Since that initial investigation, the volume of clinical and basic research pertaining to Hcy has grown exponentially [24].

To be used in clinical practice, biomarkers must be specific to cardiac injury; they should forecast future cardiovascular events and mortality rates within the general population; they need to demonstrate responsiveness, showing decreased levels after intervention and treatment; they should correlate with a risk reduction; and they must provide a low cost per quality-adjusted life year achieved [17]. Many studies have investigated cardiovascular biomarkers for risk assessment, focusing on those related to myocardial stretch (like natriuretic peptides), inflammation (emerging biomarkers), and myocyte injury (Tn). Biomarkers can be used alone or with other parameters in stratification charts, aiding in risk recalibration, early diagnosis, optimal treatment selection, and potentially preventing adverse patient outcomes. However, understanding each biomarker’s pathophysiology, analytical performance, and variability factors is essential [17].

## 2. Troponins as Cardiac Biomarkers

TnC belongs to the calmodulin superfamily of calcium receptor proteins [16,26,27]. It exists in two isoforms derived from genes associated with slow skeletal/cardiac (TNNC1) and fast skeletal (TNNC2) muscles [27]. Calcium binding to the TnC’s N-terminal domain causes conformational alterations in Tn, shifting tropomyosin along actin filaments, allowing myosin heads to attach to actin and initiate muscle contraction. The fast TnC isoform possesses two calcium-binding sites, while the slow/cardiac TnC isoform has one [28,29]. The primary function of TnC, acting as a calcium-sensing mechanism, involves the transmission of calcium signals to other Tn subunits, which in turn influence the length–tension relationship [30,31].

TnI inhibits actomyosin ATPase, significantly contributing to muscle relaxation when cytosolic calcium levels fall. TnI is produced by three unique isoform genes that are selectively expressed in slow and fast skeletal (TNNI1 and TNNI2) and cardiac (TNNI3) muscle tissues [32,33]. The C-terminal and central regions of TnI are conserved, whereas cardiac TnI (cTnI) features a distinct N-terminal extension absent in the skeletal muscle isoforms [34]. Fast skeletal muscle TnI is found in fast-twitch muscles, exhibiting reduced calcium sensitivity compared to slow TnI. This indicates that fast skeletal muscle maintains a lower activation state than cardiac and slow skeletal muscle sarcomeres [35]. The embryonic heart, in contrast to the adult heart, expresses slow skeletal muscle TnI, which provides increased calcium sensitivity and resistance to acidic pH levels. This characteristic suggests it is better equipped to withstand exercise-induced physiological stress and acidosis, similar to slow skeletal muscle [36]. The transition to cTnI occurs in the developing human heart approximately 20 days post-birth, coinciding with the cessation of hypoxia and acidosis in the fetal heart [37].

TnT is the tropomyosin-binding component of the Tn molecule, anchoring the Tn complex to the thin filament. It transmits calcium-dependent conformational alterations in TnC, thereby modulating the actin thin filament’s configuration and regulating muscle contraction and relaxation [38]. Three distinct genes are responsible for encoding the fiber-type-specific TnT proteins: TNNT1, which is associated with slow skeletal muscle; TNNT2, which pertains to cardiac muscle; and TNNT3, which is linked to fast skeletal muscle [39]. While the N-terminal region exhibits considerable variation among isoforms, the middle and C-terminal regions of TnT are notably conserved [40]. The alternative splicing of several exons contributes further to the diversity in the structure and function of TnT [39].

Cardiac troponins cTnT and cTnI are now the primary cardiovascular-specific blood biomarkers used in clinical settings. The main reasons for this are the following: First, they have significantly accelerated the diagnosis of acute chest pain, offering a dependable method for accurately diagnosing acute coronary syndromes (ACSs). Second, they have demonstrated pathological increases in various diseases, and they are recognized as independent prognostic indicators in multiple cardiovascular conditions, including ACS, chronic CAD, acute and chronic HF, along with cardiotoxicity, in addition to non-cardiovascular issues like chronic kidney disease [17]. High-sensitivity assays for cardiac troponin determination (hs-cTn) can successfully identify trace amounts of cardiac troponins TnI or TnT [41]. For an assay to be categorized as highly sensitive, it must exhibit the ability to detect cardiac troponin (cTn) in the blood of more than 50% of asymptomatic individuals while preserving a coefficient of variation below 10% at the 99th percentile among healthy individuals [42]. These analytical features lead to three significant implications. Firstly, hs-cTn assays quickly detect Tn elevation, facilitating the time of diagnosis, or it can be an exclusion criterion for ACS through rapid 1 or 2 h protocols [43,44]. Additionally, these assays can detect minor myocardial damage related to various cardiac and extracardiac conditions, providing risk stratification tools for many cases [45]. Finally, hs-cTn assays are fundamentally designed to identify minimal cTn levels in most of the general population, thereby validating cTn as a biomarker with a clearly defined normal range. This makes hs-cTn useful in the assessment of cardiovascular risk [46]. The presence of cTn in the bloodstream may arise from reversible injury linked to a normal myocyte turnover, cellular proteolytic degradation, increased permeability of the cellular wall, or membranous blebbing. Conversely, the pathways that lead to permanent injury are typically linked to tissue necrosis due to hypoxia and apoptosis. Once released into circulation, various enzymes facilitate the breakdown of cTn into smaller fragments, normally ranging from 12 to 23 kD. One way of removing proteolysis products is through the body’s reticuloendothelial system via endocytosis, and the other is by renal excretion [17,47]. While all three troponins can be found in the cardiac tissue, only cTnI and cTnT are specific to the heart. Because of that, the cTn measurement represents the gold standard for diagnosing patients with chest pain in the Emergency Department. Moreover, it is used to classify MI, as described in the Fourth Universal Infarction Consensus Document 2018 [48]. Notably, the TROPIC study found that peak levels of both cTn assays can predict events within 30 days, with cTnI potentially offering greater accuracy than cTnT in a cohort of one million patients. Furthermore, the focus on chronic kidney disease patients, regardless of their dialysis status, showed that elevated levels of cTnT and cTnI, in the absence of ACS, were linked to a poorer prognosis [17]. An important aspect to examine is understanding the elements that lead to reduced cTn levels in the blood of asymptomatic individuals. This could be caused by mechanical distension due to preload [49], myocardial remodeling or regeneration [50], stimulation of the renin–angiotensin–aldosterone systems (RAASs) or adrenergic system [51], and may also indicate the presence of subclinical CAD [52].

### 2.1. Pathophysiology of cTns in Myocardial Injury

Over 90% of cTn isoforms are found in the sarcomere; the rest remain unbound in the cytoplasm. The delivery of cTn into the blood is initiated by cellular changes, such as necrosis and programmed cell death (apoptosis), as well as increased cell membrane permeability and the subsequent release of Tn proteolysis products [53,54]. Recent findings indicate that cTn may be released without myocardial ischemia and necrosis, as evidenced by several proposed mechanisms. Under pressure or volume overload conditions, cardiomyocytes may undergo mechanical stretching, which could activate intracellular proteases that lead to the degradation of cTn. Moreover, it has been noted that tachycardia can promote cTn degradation by activation of stress-responsive integrins in cardiomyocytes without cardiomyocyte necrosis [55]. Additionally, cTn release has been observed in vivo among patients undergoing reversible ischemia during nuclear perfusion imaging with stress testing. Employing an ultrasensitive cTn I assay based on single-molecule counting technology demonstrated that fluctuations in cTn levels following stress testing were correlated with the severity of myocardial ischemia [56].

**Figure 1:**
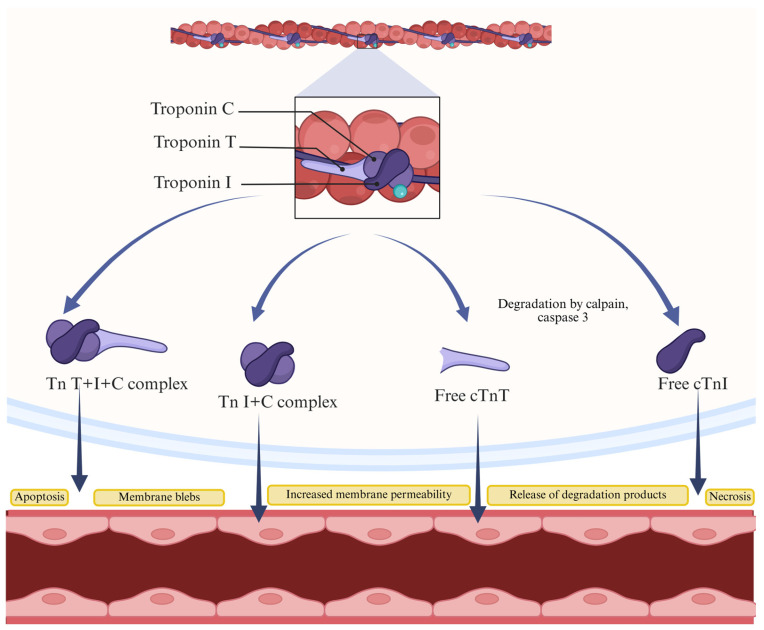
Possible mechanisms of cTn release during myocardial injury. Cardiac Tns can be released into the bloodstream as a complex of cTns (TnT, TnI, and TnC, or TnI and TnC), intact cTns (TnT or TnI), or their degradation products. The release of these substances is a consequence of various mechanisms, including the formation of membrane blebs, increased membrane permeability, necrosis, or apoptosis during myocardial injury.

Cardiac Tn release is induced by necrosis and apoptosis (Figure 1) [57,58,59]. Apoptosis of cardiomyocytes includes an increase in the activity of the caspase enzyme, which can damage DNA and impair protein structures. While necrosis impairs membrane integrity, and the cell content goes into the extracellular space, apoptosis maintains the integrity of the cell [58].

Myocardial cell regeneration could also influence cTn release. Researchers using labeled radioisotopes (14C) in myocardial cell DNA have shown that cardiomyocytes regenerate, decreasing renewal rates with age. For those 25 years old or younger, the annual renewal rate of cardiomyocytes is 1%, in contrast to a reduced rate of 0.45% for those aged 75. The authors estimate that about half of human cardiomyocytes are renewed over a lifetime [60]. They suggest this regeneration is associated with cTn release from cardiac cells into the blood, although the exact mechanism is unknown. cTn can be released from senescent or necrotic cells, which helps to clarify why healthy individuals exhibit serum cTn levels below the 99th percentile [58,60,61,62].

Cardaic Tn release could be caused by increased permeability of cardiomyocytes. Cardiomyocyte membrane permeability may be enhanced through two primary mechanisms: (1) the degradation of cell membranes by proteolytic enzymes, whose activity can elevate even during short-term myocardial ischemia; (2) due to the expansion of myocardial tissue [58]. Transient myocardial ischemia can occur during intense exercise, stress, sepsis, or ischemic heart disease. The increase in cTns is influenced by the severity of the underlying conditions [58]. The release of cTn may also result from heightened myocardial stress and distension, as research indicates a correlation between myocardial overload and elevated cTn levels. Myocardial distension in HF induces the release of natriuretic peptides. In HF, cTn and natriuretic peptide levels are influenced by the stage of this condition, thereby impacting patient prognosis according to the concentrations of these biomarkers [55,58,63].

Evidence suggests a possible mechanism for releasing cTn molecules via vesicles, as indicated by the presence of membrane vesicles on the surfaces of cardiomyocytes, with a notable increase in their number during ischemia [58,64]. In acute MI, the early surge in cTn levels is caused by cTns, while the later rise is due to the damage to the membrane of cardiac cells and the degradation of cTns in the sarcomere [58].

The size of a molecule is crucial for its capacity to traverse cell membranes, resulting in enhanced transport efficiency of low molecular weight substances. For instance, myoglobin is a small molecule that appears in the blood in the early stage of acute MI. Additionally, intracellular proteolytic enzymes significantly influence the release of cardiac marker proteins by breaking them into smaller fragments that can pass through cell membranes. Their activity can be influenced by myocardial stress, changes in cell pH, and certain medications [58,65,66].

### 2.2. Evolution of High-Sensitivity Assays

#### 2.2.1. cTnI Assays

In the 1980s, research teams began investigating cTns as specific heart condition biomarkers [1]. In 1987, Cummins developed the first radioimmunoassay (RIA) for cTnI in serum, using polyclonal rabbit antiserum. This RIA was performed within two days with a minimum detectable level of 10 ng/mL. Cummins’ study found that serum cTnI levels increased within 4 to 6 h in AMI patients, peaking at an average of 112 ng/mL at 18 h and remaining elevated for up to 8 days post-injury [67]. Three years later, two research teams [68,69] reported monoclonal antibodies targeting cTnI, with one developing an enzyme-linked immunoassay (ELISA) for serum cTnI quantification. Bodor’s assay detected concentrations as low as 1.9 μg/L and ranged up to 100 μg/mL, taking 3.5 h to complete. This cTnI assay showed high specificity for cardiac injury, even in acute and chronic muscle diseases, chronic renal failure, and post-marathon running [70]. Over the past two decades, the cTnI immunoassay has undergone significant optimization. The latest generations of commercially available assays exhibit an analytical sensitivity of one hundred times greater (1 vs. 100 ng/L) compared to the initial experimental and commercial assays. At that time, these assays lacked complete standardization, and research has shown considerable variations among different methods [71,72].

#### 2.2.2. cTnT Assays

Katus and his team developed the first immunoassays for the detection of cTnT in 1989, using an ELISA format with a biotin-linked capture antibody (M7) and a horseradish peroxidase-linked detection antibody (lBIO) [73]. Automated in 1992 via ES analyzers (Boehringer Mannheim TM) [74], this assay faced two significant issues. The first was its formulation: the capture antibody was cardiac-specific (less than 0.5% cross-reactivity to skeletal muscle), while the detection antibody had only 78% cardiospecificity. Due to 20% cross-reactivity, TnT levels were falsely elevated in severe rhabdomyolysis cases. In 1997, second-generation TnT antibodies (M11.7 used for capture and M7 used for detection) were introduced to prevent non-specific binding to skeletal TnT [75]. The second-generation assay established a normal cTnT range of 0 to 0.1 μg/L, with a limit of detection (LoD) of <0.05 and linearity up to 12 μg/L. A significant issue was the ES analyzer’s turnaround time of over 90 min, making it unsuitable for emergencies. This was resolved by using Elecsys TM analyzers, which reduced the cTnT test time to 9–18 min. Unlike the ES analyzer, Elecsys analyzers employ electrochemiluminescence immunoassay (ECLIA) technology with a ruthenium-labeled component instead of horseradish peroxidase [1]. The ‘third generation’ of the Tn T assay in 1999 represented notable progress. This generation distinguishes itself from the second by employing human recombinant cTnT for calibration instead of bovine cTnT, thereby significantly improving the assay’s linearity [76,77]. The high-sensitivity cTnT (hs-cTnT) assay is an advancement of the fourth-generation assay from 2010 [78]. In this fifth-generation version, the capture antibody remains the same, but the detection antibody is now a genetically modified mouse–human chimeric type, reducing interference from heterophilic antibodies. Improvements in analytical sensitivity were achieved by increasing the sample volume to 50 μL, raising the ruthenium concentration, and optimizing the buffer to lower background signals. As a result, the hs-cTnT assay has significantly improved, with a LoD of 5 ng/L, a 99th percentile cutoff of 14 ng/L, and a coefficient of variation of 10% at 13 ng/L [1,78].

### 2.3. Role in Diagnosing Cardiovascular Diseases

ACS presents a variety of clinical manifestations, from cardiac arrest and instability leading to cardiogenic shock to asymptomatic cases. The key symptom prompting diagnosis and treatment in suspected ACS patients is acute chest discomfort, typically described as pain, pressure, tightness, or burning. At the myocardial level, it is identified as cardiomyocyte necrosis, normally linked to non-ST-segment elevation myocardial infarction (NSTEMI), or, in rare cases, myocardial ischemia that occurs without cellular damage, referred to as unstable angina [44].

Advanced cTn tests and lower diagnostic thresholds have updated guidelines for detecting myocardial injury. The fourth universal definition of MI sets a global standard for classifying myocardial injury and infarction [48,54]. The fourth universal definition of MI updated the concepts of MI types, as follows:

Type 1 MI: Focus on the link between plaque disruption and coronary atherothrombosis.

Type 2 MI: Occurs when there is an imbalance between oxygen demand and supply, not associated with acute coronary atherothrombosis.

Type 2 MI: The significance of CAD presence or absence concerning prognosis and treatment.

Type 3 MI: Explanation of the importance of distinguishing Type 3 MI from sudden cardiac death.

Types 4–5 MI: Highlighting the importance of distinguishing between myocardial injury due to procedures and MI resulting from procedures [48].

The clinical criteria for MI indicate the occurrence of acute myocardial injury, which is identified through abnormal cardiac biomarkers alongside evidence of acute myocardial ischemia. The criterion for identifying myocardial injury is established when the cTn value exceeds the 99th percentile upper reference limit (URL). This injury is classified as acute if there is a fluctuation in cTn values, either an increase or a decrease [48]. Type 1 MI is detected by a rise and/or fall in cTn values, with at least one measurement above the 99th percentile URL, along with at least one of the following: acute myocardial ischemia symptoms; new ischemic changes on an ECG; pathological Q waves; imaging showing a new loss of myocardium or local wall motion abnormalities; or coronary thrombus identified via angiography or autopsy [48]. To detect Type 2 MI, there must be an increase and/or fall in cTn levels, with at least one measurement above the 99th percentile URL, and evidence of a mismatch between myocardial oxygen supply and demand not due to acute coronary atherothrombosis. This requires at least one of the following: new ischemic changes on an ECG; symptoms of acute myocardial ischemia; pathological Q waves; or imaging showing new loss of myocardium or new local wall motion abnormalities consistent with ischemia [48]. The Type 3 MI criteria include patients who die from cardiac causes with symptoms of myocardial ischemia and new ischemic ECG changes or ventricular fibrillation, but who die before blood samples for biomarkers can be taken or before cardiac biomarkers rise or before autopsy confirms MI [48]. Cardiac procedural myocardial injury is indicated by cTn values exceeding the 99th percentile URL in patients with normal baseline values. It can also be defined as a rise in cTn values greater than 20% from the baseline when the baseline is above the 99th percentile URL, as long as the values are stable or declining [48]. The Type 4a MI criteria within 48 h post-procedure include a rise in cTn levels exceeding five times the 99th percentile URL for patients with normal baseline values. For those with stable or decreasing elevated pre-procedural cTn levels (variation of 20% or less), the post-procedural cTn must increase by over 20% and still exceed five times the 99th percentile URL. Additionally, at least one of the following must be present: new ischemic changes on ECG; new pathological Q waves; imaging showing new loss of myocardium or local wall motion abnormalities; or angiographic evidence of complications affecting flow, such as coronary dissection or major artery occlusion [48]. Type 5 MI related to CABG is characterized by a rise in cTn levels exceeding 10 times the 99th percentile URL in patients with normal baseline cTn. If preoperative cTn levels are elevated but stable (within 20% variation) or decreasing, post-operative cTn must increase by over 20% and still exceed 10 times the 99th percentile URL. Additionally, at least one of the following must occur: new pathological Q waves, angiographic evidence of new graft or coronary artery occlusion, or imaging showing new loss of myocardium or regional wall motion abnormalities indicative of ischemia [48].

Unstable angina, recognized as an entity within ACS, involves myocardial ischemia at rest or with minimal exertion without acute cardiomyocyte injury. In a study of patients with suspected non-STEMI-ACS, using hs-cTn instead of standard assays increased MI detection by 4% and reduced unstable angina diagnoses. Unlike NSTEMI patients, those with unstable angina show no acute injury or necrosis, have a lower mortality risk, and benefit less from aggressive antiplatelet therapy and invasive treatments within 72 h [44]. Findings from a substantial cohort of real-world patients indicate that the diagnosis of unstable angina remains prevalent despite advancements in cTn assays that offer enhanced sensitivity. The percentage of true cTn-negative unstable angina among individuals hospitalized with non-ST-elevation ACS is between 5% and 6% [79].

Apart from predicting new cases of HF [80], the rise in high-sensitivity cardiac Tn (hs-cTn) is associated with adverse outcomes in individuals with established HF. Patients with acute and chronic HF often exhibit elevated hs-cTn levels, which may yield supplementary prognostic data in both contexts [81,82]. Measuring cTn is recommended for evaluating acute HF patients to detect Type 1 MI as a possible cause. However, the frequent elevation of hs-cTn complicates interpretation in these patients. If Type 1 MI is absent, cTn changes may arise from several factors, including Type 2 MI from supply–demand issues and non-coronary myocardial injury. Cardiac Tn levels can also increase due to elevated preload, even without ischemia [83]. Regardless of the acute HF cause, increased hs-cTn concentrations predict risk [82]. An analysis of ten studies indicated that hs-cTn is a standalone predictor of cardiovascular-related hospitalizations, cardiovascular death, and total mortality, regardless of the presence of CAD [84].

Hs-cTn has shown prognostic value in individuals with valvular heart disease, especially in those suffering from aortic stenosis. Consequently, hs-cTn is utilized to identify asymptomatic patients with aortic stenosis and risk of myocardial fibrosis for participation in a trial assessing the impact of early aortic valve replacement in this demographic, particularly focusing on those with mid-wall late gadolinium enhancement [85].

### 2.4. Role of Cardiac Troponins in Risk Stratification and Prognosis in Patients with Cardiovascular Diseases

One advantage of hs-cTn assays is their ability to accelerate the assessment of patients suspected of having ACS, primarily due to the prompt recognition of low-risk individuals who can be discharged early. This alleviates overcrowding in the emergency department while not escalating resource utilization [86]. Individuals experiencing acute chest pain with 30-day mortality or a major adverse cardiovascular events (MACEs) risk of less than 1% should be classified as low risk [87]. In cases of acute chest pain among intermediate-risk patients, it is recommended to utilize transthoracic echocardiography (TTE) as a prompt bedside assessment to establish baseline ventricular and valvular function, check for wall motion abnormalities, and assess the presence of pericardial effusion [87]. Patients experiencing acute chest pain with suspected ACS who exhibit new ischemic changes on an electrocardiogram, cTn-confirmed acute myocardial injury, newly occurred left ventricular systolic dysfunction with ejection fraction < 40%, recently identified moderate to severe ischemia during stress testing, hemodynamic instability, and/or a high clinical decision pathway (CDP) risk score should be classified as high risk for short-term MACE [87].

According to the 2021 AHA/ACC guidelines, a class 1B recommendation (Level of Evidence B-NR [nonrandomized]) indicates that in cases of acute chest pain with suspected ACS, CDPs should sort patients into low-, intermediate-, and high-risk groups to enhance their management and subsequent diagnostic processes [87]. The low-risk category is clearly defined, whereas the criteria for the intermediate- and high-risk categories are less precise. Individuals may be classified as intermediate risk solely based on a risk score, even if their hscTn levels are below the 99th percentile. Nevertheless, in many cases, determining myocardial injury, indicated by cTn levels above or below the 99th percentile, is a crucial factor in predicting adverse outcomes [48,86].

Much evidence has demonstrated the significance of hs-cTnI in cardiovascular risk assessment among seemingly healthy or asymptomatic individuals [41]. The suggested thresholds for employing high-sensitivity cTn I in the context of cardiovascular risk assessment in asymptomatic individuals are as follows: (1) low cardiovascular risk: male < 6 ng/L, female < 4 ng/L; (2) moderate: male 6–12 ng/L, female 4–10 ng/L; (3) high: male > 12 ng/L, female > 12 ng/L [88].

## 3. Hcy as Cardiovascular Biomarker

**Figure 2:**
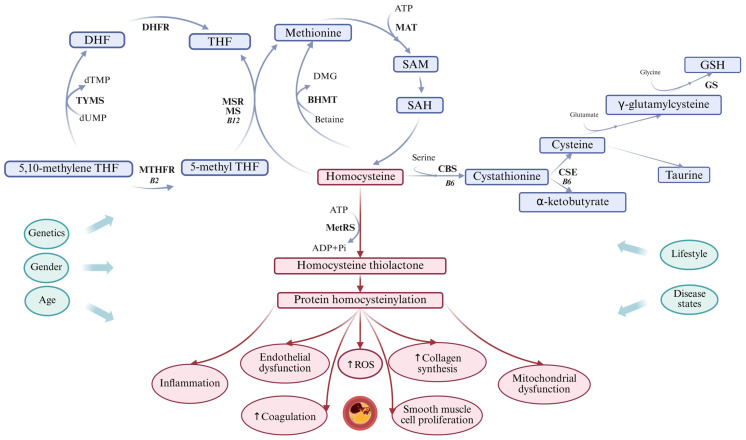
Hcy metabolism. Hcy metabolism involves remethylation to methionine and transsulfuration to cysteine (presented by blue arrows). Protein homocysteinylation can lead to cardiovascular diseases (presented by red arrows) and is influenced by various non-modifiable risk factors, such as genetics, gender, and age, as well as modifiable risk factors, including lifestyle and disease states (presented by green arrows). B2—Vitamin B2, B6—Vitamin B6, B12—Vitamin B12, CBS—Cystathionine β-synthase, CSE—Cystathionine γ-lyase, DHF—Dihydrofolate, DHFR—Dihydrofolate reductase, DMG—Dimethylglycine, dTMP—Deoxythymidine monophosphate, dUMP—Deoxyuridine monophosphate, GS—Glutathione synthase, GSH—Reduced glutathione, MAT—Methionine adenosyltransferase, MetRS—Methionyl-tRNA synthetase, MTHFR—Methylenetetrahydrofolate reductase, ROS—Reactive oxygen species, SAH—S-adenosyl Hcy, SAM—S-adenosyl methionine, THF—Tetrahydrofolate.

Hcy is a non-essential, sulfur-containing, non-proteinogenic amino acid. It is synthesized through the transmethylation of methionine (Met), which is an essential amino acid obtained from dietary sources. This process, illustrated in Figure 2, represents the only pathway for Hcy production in humans. The human body cannot absorb Hcy from food or produce it on its own. Met is found in various foods, including poultry, meat, eggs, seafood, and dairy products. In the synthesis of Hcy, methionine is converted by removing the terminal methyl group. This conversion involves three steps, catalyzed by the following enzymes: *S*-adenosyl-L-methionine (SAM) synthetase, methyltransferase (MT), and *S*-adenosyl-L-Hcy (SAH) hydrolase. SAM synthetase activates Met in a reaction with ATP, leading to the synthesis of SAM. SAM serves as a universal methyl donor and plays a crucial role in various cellular biosynthetic processes. It is involved in epigenetic modifications, including the regulation of DNA methylation, chromatin remodeling, RNA editing, noncoding RNA functions, microRNA, and the post-translational modification of histones. The byproduct of all SAM-dependent transmethylation reactions is SAH. Hcy serves as a branching point for three major pathways, predominantly located in the liver: (a) resynthesis to SAH via the reverse activity of SAH hydrolase; (b) remethylation to methionine through folate and B12-dependent or independent pathways; and (c) transsulfuration, which involves two enzymes: cystathionine β-synthase (CBS) and cystathionine γ-lyase (CSE), crucial for the conversion of Hcy into cysteine via the intermediate metabolite cystathionine [89,90]. Under normal physiological conditions, the production and transformation of Hcy are carefully regulated. Hcy is produced in various tissues, with the liver being the primary site for these processes. Therefore, it is reasonable to hypothesize that liver impairment may result in changes to Hcy levels [91].

The concentration of Hcy in the blood is typically measured as the total Hcy concentration (tHcy). Most of the Hcy in plasma combines with other substances to form compounds, such as mixed Hcy disulfides and homocysteinylated proteins, while only a small fraction exists as free monomers. Hcy is found in both reductive (1%) and oxidative (99%) forms in the blood. The oxidative forms (*S*-homocysteinylated and *N*-homocysteinylated proteins) are also divided into three subtypes: disulfide Hcy-Hcy (Hcy-SS-Hcy, 5–10%), disulfide Hcy-cysteine (Hcy-SS-Cy, 5–10%), and protein-bound Hcy (*N*-linked and *S*-linked to *γ*-globulins or albumins, 80–90%) [24,92].

Researchers have been puzzled by the varying “normal” levels of tHcy for an extended period. This confusion arises from several factors, including differing criteria for patient inclusion, variations related to race and geographic location, genetic differences, and various health risk factors. Additionally, inconsistencies may emerge from how samples are collected and tested. Over the past two decades, plasma Hcy concentrations have been classified into four categories: normal (5–15 µmol/L), moderate (>15–30 µmol/L), intermediate (31–100 µmol/L), and severe (>100 µmol/L) [7,8]. Typically, older reference values for Hcy are higher than those suggested in recent guidelines. Nevertheless, some researchers argue that normal plasma Hcy concentrations should not dip below 6.3 µmol/L. Drawing from substantial evidence, Wu et al. recommend a normal reference range for plasma Hcy levels between 5 and 10 µmol/L, which is more reasonable and beneficial for detecting, preventing, and treating HHcy [24].

CVDs account for nearly one-third of all global deaths [93]. Therefore, understanding the pathogenesis and treatment of HHcy is of significant clinical importance. The causal relationship between HHcy and the incidence of CVDs cannot be overstated. The concentration of HHcy can be effectively reduced through simple, safe, and cost-effective interventions. It is imperative to comprehend the etiology of HHcy in order to develop targeted interventions.

Elevated levels of Hcy are influenced by a complex interplay of dietary practices, genetic polymorphisms, and metabolic conditions [23]. Folic acid, taurine, and omega-3 fatty acid supplementation have been shown to mitigate some adverse effects associated with high Hcy levels. The deficiencies of these nutrients correlate directly with HHcy, highlighting the importance of nutritional status in regulating Hcy [94,95,96]. Zhang et al. highlight the potential of metabolomics in identifying novel biomarkers and elucidating pathways involved in Hcy metabolism and its implications for cardiovascular health [97]. Addressing these determinants through targeted dietary interventions, genetic counselling, and metabolic management is essential for mitigating the risk of CVDs and other associated health complications, thereby also contributing to the deceleration of the ageing process.

### 3.1. Toxicity of Hcy and Related Compounds

Over the years, several hypotheses regarding Hcy toxicity have been developed. However, despite ongoing research, none of these explanations fully clarifies the mechanisms behind Hcy biotoxicity. The literature discusses three primary pathways [90] through which Hcy exerts its toxic effects, as follows: (1) modifications to protein structure, referred to as homocysteinylation; (2) the induction of oxidative stress; (3) excitotoxicity.

Hcy toxicity is believed to result from the covalent binding of this compound to proteins, which subsequently alters their functions. This process, known as homocysteinylation, is regarded as a post-translational modification of proteins. The extent of protein homocysteinylation is directly proportional to the increased levels of plasma Hcy [98].

We thoroughly reviewed the relevant literature and determined that oxidative stress and the activity of free radical reactions have been proposed as mechanisms underlying the pathophysiological effects of Hcy. Oxidative stress, resulting from the buildup of reactive oxygen species (ROS), is the main mechanism behind Hcy-induced vascular injury. In addition to forming a disulfide bond with cysteine, Hcy also directly inhibits the activity of antioxidant enzymes [99].

Various mechanisms have been suggested to clarify how Hcy contributes to the induction of oxidative stress: (a) Hcy autoxidation; (b) inhibition of antioxidant enzyme activity in cells; (c) disruption of extracellular superoxide dismutase on endothelial surfaces; (d) activation of NADPH oxidases; and (e) nitric oxide synthase (NOS)-dependent generation of superoxide anion [100]. Additionally, Hcy has been found to increase the expression of methylase and upregulate the methylation of glutathione peroxidase 4 (GPX4), leading to oxidative stress and ferroptosis in the nucleus pulposus, an occurrence that requires further investigation [101,102]. Moreover, oxidative stress contributes to the formation of nitrotyrosine, which is an indicator of the reaction between nitric oxide and superoxide radicals. This reaction produces peroxynitrite, a potent oxidant. Peroxynitrite can lead to the nitration of tyrosine, resulting in changes to protein function and ultimately causing cellular dysfunction [103].

Oxidative stress and the activity of free radical reactions have been demonstrated to induce endoplasmic reticulum (ER) stress and to activate the unfolded protein response (UPR) [104,105]. ER stress plays a significant role in the development of atherosclerosis [104]. Simultaneously, the activation of the CHOP/GADD153 and T-cell death-associated protein (TDAG-51) pathways, both essential UPR routes, leads to the impairment of endothelial integrity through endothelial cell apoptosis and anoikis [106].

Furthermore, research has demonstrated that HHcy preferentially triggers pyroptosis in vascular endothelial cells through the activation of caspase-1-dependent inflammasomes, resulting in endothelial dysfunction. Recent studies have highlighted the significance of pyroptosis, a type of programmed cell death, in the development of atherosclerotic plaques [101]. Hcy can accelerate atherosclerosis by inducing pyroptosis in macrophages through various mechanisms, such as ER stress and calcium imbalance [97].

The cell death caused by HHcy is primarily due to the presence of Hcy itself, and it is not exclusively governed by oxidative stress mechanisms. Interestingly, this type of cell death can also be mimicked by various agents that activate the UPR. The UPR is a crucial signaling pathway that comes into play when there is an accumulation of misfolded or unfolded proteins within the ER. This indicates that the mechanisms behind cell death in the context of HHcy are more complex and involve a distinct interplay of cellular stress responses [107].

In the context of Hcy-induced cell death, the activation of the UPR triggers a cascade of intracellular events that ultimately lead to programmed cell death or apoptosis. This intricate process begins with the activation of sensors located in the ER membrane, one of the key players being the inositol-requiring enzyme 1 (IRE1). Upon activation, IRE1 performs a crucial function by splicing the mRNA of X-box binding protein 1 (XBP1). This splicing results in the production of a highly potent transcription factor known as spliced XBP1 (XBP1s). The emergence of XBP1s plays a significant role in orchestrating the cellular machinery that drives the final stages of apoptosis, marking the cell’s irreversible commitment to death in response to the stress induced by elevated levels of Hcy [108].

### 3.2. Dysregulated Hcy Levels and Cardiovascular Diseases

Currently, the relationship between imbalances in Hcy metabolism and various pathological conditions is still not well understood. By the 1990s, a surge of studies investigating the hypothesis highlighted HHcy as a significant risk factor for CVDs. Today, it is widely accepted that an elevated Hcy level is an independent cardiovascular risk factor. Increased serum levels of Hcy have been linked to a higher risk of premature arteriosclerosis and CVDs [109,110,111]. Research indicates that a rise of 2.5 µmol/L in plasma tHcy concentrations is linked to a 10% increase in CVD risk [112]. These conditions encompass hypertension [24,113,114], CAD [115,116,117,118,119], HF [120], stroke [121,122], aortic dissection [123], and venous thrombosis [124].

Previous studies have indicated that high Hcy levels are independent risk factors for atherosclerosis and CAD. HHcy (above 20 µmol/L) is associated with a nine-fold increase in the risk of MI and subsequent stroke compared to concentrations below 9 µmol/L [125]. Hcy influences the severity of coronary lesions through multiple mechanisms, including direct toxicity to the vascular endothelium, promotion of thrombosis, and modifications to vascular structure and function. Furthermore, elevated Hcy levels correlate with the instability of coronary artery plaques [126]. A significant issue that continues to require clarification is the extent to which HHcy directly contributes to the development of vascular diseases, as opposed to serving merely as a biomarker that indicates other metabolic changes affecting vascular function. Research has shown a positive correlation between elevated Hcy levels and the incidence of MI, even after accounting for additional CVD risk factors. This compelling connection suggests that Hcy might emerge as the “cholesterol” of the 21st century, warranting greater attention in clinical settings [90]. The connection between CAD and HHcy not only offers important insights for diagnosing CAD but may also act as a potential prognostic indicator for predicting disease outcomes [127].

The effect of elevated Hcy levels on endothelial cell health may contribute to the development of hypertension, as increased circulating Hcy was linked to greater arterial stiffness in prehypertensive patients [128]. Research indicates that HHcy is independently linked to isolated systolic hypertension and may reduce the effectiveness of angiotensin-converting enzyme (ACE) inhibitors in patients with hypertension. This implies that Hcy negatively impacts both the mechanisms that contribute to hypertension and the efficacy of antihypertensive treatments [24]. Hcy is implicated in endothelial dysfunction. It reduces the bioavailability of nitric oxide (NO) by inhibiting endothelial nitric oxide synthase eNOS and increasing the levels of asymmetric dimethylarginine (ADMA), an endogenous inhibitor of NO synthase. This leads to vasoconstriction and a decrease in coronary blood flow [99]. Additionally, the autooxidation of Hcy induces the production of ROS, damages the endothelium, and triggers inflammation [129]. It also promotes the expression of adhesion molecules such as vascular cell adhesion molecule 1 (VCAM-1), intercellular adhesion molecule 1 (ICAM-1), and E-selectin, along with pro-inflammatory cytokines, thereby exacerbating the process of atherosclerosis [130]. Vascular dysfunction can lead to the release of cTn through several mechanisms, including the following: (a) coronary microvascular ischemia [131], (b) procoagulant effects and microvascular embolism due to platelet activation [132], and (c) chronic endothelial dysfunction caused by remodeling, which is characterized by fibrosis and arterial stiffness [133].

Recently, it has been reported that the SAM/SAH ratio may serve as a biomarker, providing a sensitive indicator for the clinical diagnosis of atherosclerosis [134].

Another research group investigated the effects of elevated Hcy levels on fatty acid-binding protein 4 (FABP4). Their findings revealed that FABP4 plays a crucial role in lipid metabolism disturbances following Hcy treatment. Additionally, they suggested that DNA methyltransferase 1 could be a potential therapeutic target for Hcy-related atherosclerosis [135].

While aortic arterial dissection, a severe acute vascular condition characterized by high mortality rates and poor prognosis, has also been reported in connection with HHcy, the precise mechanisms underlying this relationship remain unclear [123].

A Mendelian randomization study examined the causal relationship between Hcy levels and the risk of congestive HF and cardiomyopathy. The findings of Mendelian randomization studies do not offer sufficient evidence to negate the pathogenic effects of Hcy on vascular injury [136].

## 4. Clinical Applications: Integrating Biomarkers for Improved Cardiovascular Risk Prediction

While previous sections have addressed the molecular mechanisms involving Tns and Hcy, this section synthesizes current clinical evidence regarding their prognostic and diagnostic roles in cardiovascular risk assessment.

CVDs remain the leading cause of mortality globally, with CAD comprising the predominant subset among all cardiovascular conditions. According to data from 2021, CVD accounted for 20.5 million deaths, representing nearly one-third of total global mortality, and projections suggest that this number could reach 23.6 million by 2030 [137,138]. Contemporary epidemiological trends demonstrated a shift in the age distribution of affected individuals, with an increasing proportion of cases occurring in patients younger than 65 years. This trend can be largely explained by the growing prevalence of metabolic disorders, such as diabetes mellitus (DM) and obesity, alongside sustained exposure to modifiable risk factors, particularly tobacco use. Given the significant public health burden posed by CVD, improving existing and developing novel strategies for early detection and cardiovascular risk assessment is essential in enabling more effective prevention and timely therapeutic intervention [139].

Traditional approaches to cardiovascular risk stratification are based on well-established risk factors for atherosclerotic disease, with numerous algorithms developed according to their relative contributions. Risk assessment has predominantly relied on biomarkers derived from the “lipid hypothesis”, which posits a direct correlation between cholesterol levels, related plasma lipoproteins, and atherosclerotic risk. These variables have been incorporated into contemporary risk scoring systems, such as the Framingham Risk Score, the ASSIGN Score, the PROCAM Score, and the European Society of Cardiology’s (ESC) SCORE2/SCORE2OP. However, clinical experience demonstrates that a considerable number of patients develop cardiovascular events despite optimal management of recognized risk factors, suggesting the presence of residual risk that is not adequately captured by standard assessment tools [140,141].

Despite the availability of established cardiovascular risk assessment algorithms, a clear need for more precise diagnostic tools to enhance clinical decision-making remains. The integration of additional biomarkers may facilitate the identification of high-risk patients who are not adequately recognized by conventional methods, while also reducing unnecessary diagnostic and therapeutic interventions in low-risk individuals. An ideal biomarker should be specific to cardiac pathology, detectable in asymptomatic individuals, associated with long-term cardiovascular outcomes, and modifiable through preventive strategies, all while offering incremental prognostic value beyond current risk models [41,46]. In this context, biomarkers facilitate more precise patient stratification, allowing for the optimization of therapeutic strategies while minimizing unnecessary treatment. This approach represents a critical advancement toward personalized medicine in cardiovascular care, enabling the customization of preventive and therapeutic interventions based on individual patient characteristics [142].

cTns are considered the gold standard among circulating biomarkers for detecting myocardial injury and cardiomyocyte necrosis, serving as key reference parameters in the evaluation of cardiovascular pathology [143]. A breakthrough in the detection of myocardial damage occurred with the development of hs-cTn assays, which have significantly transformed both diagnostic and prognostic capabilities in cardiovascular medicine. The clinical value of hs-cTn stems from several important aspects. Most notably, high-sensitivity assays have greatly improved the differential diagnosis of acute chest pain by enabling accurate identification of ACS. In addition, pathological elevations of cTn levels have been documented across a broad spectrum of cardiovascular conditions, including chronic CAD, acute and chronic HF, and chemotherapy-induced cardiotoxicity. Elevated hs-cTn levels may indicate the presence of subclinical atherosclerosis and serve as a marker of increased cardiovascular risk. This enhanced ability to stratify risk facilitates the early identification of high-risk individuals who may benefit from timely preventive strategies aimed at reducing the incidence of cardiovascular events [144].

Beyond hs-cTn, recognized as a highly sensitive marker of myocardial necrosis, Hcy has gained attention as a potential biomarker of vascular dysfunction, enhancing the overall assessment of cardiovascular risk. Increased Hcy concentrations have been shown to contribute to oxidative stress, inflammation, endoplasmic reticulum stress, and apoptosis, as well as to stimulate autoimmune responses and activate the coagulation cascade, thus heightening the risk of thrombosis [145]. Over recent decades, multiple studies have consistently demonstrated a strong association between Hcy and CAD, HF, and cerebrovascular stroke [146]. According to a prospective cohort study conducted in Beijing, each 5 µmol/L increase in Hcy levels was associated with a 4% rise in cardiovascular risk and a 5% higher risk of all-cause mortality [147]. Nevertheless, despite these findings, the clinical utility of Hcy as a biomarker remains limited. While Hcy offers a valuable insight into the pathophysiological mechanisms underlying CVD, its practical application as a predictive and therapeutic marker in routine clinical practice remains the subject of ongoing investigation. Moreover, although interventional studies involving folate and B-vitamin supplementation aimed at lowering Hcy levels have yielded promising results in selected patient subgroups, their effectiveness in reducing overall cardiovascular risk has not been consistently confirmed [148]. These limitations underscore the need for further research to clarify the role of Hcy in cardiovascular pathology, as well as the potential synergistic value of combining hs-cTn and Hcy in the stratification of high-risk patients.

## 5. Research Findings: From Individual to Combined Biomarker Utility

In the field of cardiovascular medicine, current research is increasingly focused on developing more accurate risk assessment methods and identifying patients prone to complications. Although traditional risk stratification models remain fundamentally important, their integration with biomarkers of myocardial and vascular dysfunction may substantially enhance clinical decision-making. In this context, hs-cTn and Hcy emerge as potentially complementary indicators, providing a more comprehensive understanding of myocardial injury and vascular impairment [149,150].

hs-cTn assays have established themselves as pivotal biomarkers in contemporary cardiovascular practice, demonstrating both diagnostic and prognostic utility across diverse populations and clinical settings. In a meta-analysis of 28 prospective cohorts encompassing 154,052 individuals without known CVD, hs-cTnI and hs-cTnT were detectable in approximately 80% of asymptomatic participants. Those with Tn concentrations in the upper tertile of the population distribution faced a 43% higher risk of any cardiovascular event, a 59% greater risk of CAD, and a 67% increased risk of fatal cardiovascular outcomes. Additionally, a 35% rise in stroke risk was observed, likely reflecting an association with arrhythmic disorders such as paroxysmal atrial fibrillation [151]. A separate meta-analysis found that each one standard deviation increment in hs-cTn corresponded to a 23% increase in all-cause mortality, an 82% increase in cardiovascular mortality, a 33% increase in major cardiovascular events (MACEs), and a 49% increase in hospitalizations due to HF. Notably, hs-cTn exhibits a low index of individuality (~0.3), indicating that even small intra-individual changes are more likely to reflect true alterations in myocardial injury, unlike other biomarkers with a higher index of individuality [84].

As previously mentioned, the introduction of hs-cTn has fundamentally transformed the diagnostic approach to ACS, enabling rapid and precise stratification of patients with acute chest pain. Accordingly, hs-cTn has been integrated into the Fourth Universal Definition of Myocardial Infarction, as well as into the current diagnostic algorithms of the European Society of Cardiology (ESC) and the American College of Cardiology/American Heart Association (ACC/AHA) for evaluating patients with chest pain without persistent ST elevation on an ECG. The ESC algorithm using serial measurements of hs-cTnT/I at admission and after 1 h (0/1 h algorithm) combines high safety with high efficiency in early exclusion or confirmation of acute MI [152]. The APACE study, conducted on a large number of patients with acute chest pain, confirmed the high diagnostic reliability of the ESC 0/1 h algorithm based on hs-cTn, which achieved a negative predictive value greater than 99% for excluding major adverse cardiovascular events within 30 days. It is particularly significant that the algorithm demonstrated high efficiency even in patients presenting within the first two hours of symptom onset, enabling rapid and reliable triage of more than half of the patients with suspected ACS [153]. These findings were also confirmed in the TRAPID-AMI study [154].

The implementation of hs-cTn in clinical practice has led to a significant improvement in diagnostic precision for detecting AMI, resulting in the reclassification of a substantial number of patients from the unstable angina pectoris category to the NSTEMI category. This diagnostic shift has significant implications for risk stratification and therapeutic approach. The exceptional sensitivity of hs-cTn enables the identification of minimal myocardial damage that was previously below the detection threshold of conventional Tn tests, contributing to a more precise diagnostic algorithm and more adequate treatment of patients with ACS [155]. Additional evidence of the prognostic value of hs-cTn was provided by findings from a large cohort study indicating that hs-cTnI can independently predict the development of incident HF. These findings further underscore the potential of hs-cTn as a key biomarker in cardiovascular risk assessment, particularly within a multi-marker framework, where its prognostic value is most pronounced when incorporated as a central component [156]. According to findings from a systematic review with meta-analysis of 16 prospective studies, individuals with hs-cTn concentrations in the highest tertile of the population distribution exhibited a two-fold increased risk of developing HF compared to those in the lowest tertile, independent of traditional risk factors and natriuretic peptide levels. This association was consistently observed across diverse populations and in both sexes [157].

hs-cTn has also found application in the diagnosis of the cardiovascular toxicity of various etiologies. The 2022 ESC guidelines recommend measuring hs-cTn in monitoring patients undergoing potentially cardiotoxic antineoplastic therapy [158]. A recent meta-analysis confirmed the diagnostic value of elevated hs-cTn concentrations in the early detection of cardiotoxicity, during the period of 3–6 months after initiating therapy with anthracyclines, trastuzumab, and their combination, as well as immune checkpoint inhibitors or radiotherapy in combination with anthracyclines [159]. Additionally, hs-cTnT concentrations above the 99th percentile, as well as lower threshold values of 7 ng/L, have shown consistent prognostic value in risk stratification for cardiotoxicity induced by oncological therapy in various clinical contexts, including curative, adjuvant, and palliative oncology protocols, as well as in patients after the completion of active treatment [160]. The clinical significance of hs-cTn has also been evaluated in the context of cardiovascular toxicity associated with metabolic imbalance in DM. Chronic hyperglycemia, as the central pathophysiological substrate of DM, leads to progressive myocardial damage through increased oxidative stress, microvascular dysfunction, and accumulation of advanced glycation end products (AGEs) [161]. In this context, hs-cTns stand out as sensitive biomarkers of subclinical myocardial damage. The research results confirm a significant correlation between elevated hs-TnI values in patients with type 2 DM and increased risk for incident cardiovascular events, including HF, MI, and cardiovascular mortality. These findings indicate the potential of hs-cTns in the stratification of cardiovascular risk associated with chronic hyperglycemia, enabling early detection of myocardial vulnerability in asymptomatic diabetic patients [162,163].

Takotsubo cardiomyopathy, also known as stress-induced cardiomyopathy, represents a reversible form of left ventricular dysfunction that develops due to a sudden increase in circulating catecholamines. Although there is an increase in cardiac Tn concentrations, their maximum value in this syndrome usually remains lower compared to MI, despite often pronounced regional hypokinesia. Beyond quantitative differences, recent research indicates a qualitatively distinct pattern of cTn release in Takotsubo syndrome; shorter cTnT fragments predominate in circulation (>85%), unlike in MI, where longer protein forms dominate (>60%) in the early hours after symptom onset. This differential Tn expression may represent additional diagnostic value in the early clinical evaluation of these entities [164,165,166,167].

In summary, hs-cTn revolutionized cardiac diagnostics—from the precise detection of ACS, through cardiovascular risk stratification in the general population, to monitoring the cardiotoxicity of various etiologies—enabling personalized assessment and appropriate therapeutic decision-making across a wide spectrum of clinical scenarios.

In parallel with research on the prognostic value of hs-cTn, a substantial body of evidence has identified Hcy as a potential biomarker of cardiovascular risk. Although numerous studies have established Hcy as a potential biomarker of cardiovascular risk, fundamental uncertainty remains as to whether elevated Hcy levels represent a causal factor or a consequence of CVD, complicating its clinical interpretation [168]. A recent meta-analysis, which included 9381 patients with CAD and 12,188 controls from 59 studies, showed significantly higher serum Hcy concentrations in affected individuals, suggesting its association with atherosclerotic processes. Nevertheless, the pronounced heterogeneity among studies somewhat limits the reliability of these findings [145]. A large prospective Norwegian study indicated that subjects with Hcy concentrations ≥ 20 µmol/L had a 3.6 times higher risk of cardiovascular and total mortality compared to individuals with values < 9 µmol/L, further highlighting its potential prognostic significance [169]. In addition, results from contemporary research indicate that Hcy, especially in combination with traditional lipid parameters such as LDL cholesterol and the ratio of total to HDL cholesterol (TC/HDL), may represent a strong predictor of risk for developing CAD. One study highlighted a significant correlation between Hcy and certain components of the lipid profile, where multivariate regression analysis confirmed the high predictive value of these biomarkers in cardiovascular risk assessment. These findings emphasize the importance of integrating Hcy into the framework of standard cardiac evaluation as a potentially useful supplementary marker in identifying subjects with an increased risk of atherosclerotic events [116].

Additionally, a multicenter study confirmed the association of Hcy with various forms of ACS, including ST-elevation MI (STEMI) and NSTEMI [119]. Calim et al. analyzed the relationship between Hcy and the GRACE score, a validated prognostic tool for risk stratification in patients with ACS. In patients with NSTEMI, a statistically significant, moderately positive correlation was identified between Hcy concentration and the GRACE score (*p* < 0.05), while such a correlation was not found in patients with STEMI. Nevertheless, in the overall sample, a significant positive relationship between Hcy and the GRACE score was observed [115]. Beyond atherosclerosis, the significance of Hcy is also being investigated in HF. Jin et al. demonstrated that plasma Hcy concentrations are significantly higher in patients with HF compared to the control group, with a positive correlation with disease severity progression according to the NYHA classification [170]. Furthermore, Karger et al. established a significant association between Hcy levels and HF with a preserved ejection fraction, while such a correlation was not found in patients with HF with a reduced ejection fraction. These findings suggest that the mechanisms linking Hcy and HF might depend on the specific pathophysiological characteristics of different phenotypes of this disease [146]. The mentioned studies indicate a significant association of Hcy with various cardiovascular conditions, which raises questions about specific pathophysiological mechanisms in the development and progression of these diseases.

The multifaceted pathophysiological mechanisms underlying the toxic effects of Hcy, such as oxidative stress, endothelial dysfunction, prothrombotic activity, and epigenetic alterations, have been comprehensively outlined in previous sections. These interrelated pathways offer a plausible biological explanation for the consistent association between elevated Hcy levels and cardiovascular morbidity. Importantly, the particular vulnerability of cardiomyocytes and vascular structures to the harmful effects of Hcy is further amplified by their naturally lower expression of cystathionine β-synthase, a key enzyme in Hcy metabolism [171]. This vulnerability may amplify clinical consequences even in cases of moderate HHcy, underscoring the relevance of Hcy as a biomarker of subclinical vascular dysfunction.

Previous research has focused on evaluating individual biomarkers to improve cardiovascular risk assessment, with hs-cTn and Hcy identified as significant indicators of myocardial injury and vascular dysfunction. However, the predictive value of isolated biomarkers, although statistically significant, often remains limited in clinical practice, with a modest contribution to more precise risk stratification. Therefore, increasing attention is being directed toward multi-marker approaches, which involve the simultaneous determination of multiple biomolecules for a more comprehensive and precise evaluation of cardiovascular status [172]. Population studies have highlighted the value of simultaneous measurement of hsTnI, natriuretic peptides, and Hcy in cardiovascular risk assessment. While hs-TnI and natriuretic peptides are highly specific markers for cardiac pathology, Hcy shows a significant role as a complementary biomarker. The simultaneous determination of these parameters can contribute to more precise identification of individuals at increased risk for developing CVD, particularly in the context of preventive population strategies [173]. In this context, concurrent analysis of hs-cTn and Hcy represents a potentially valuable strategy, given their complementary diagnostic and prognostic roles—hs-cTn reflects myocardial damage, while Hcy reflects the degree of endothelial dysfunction and a proatherogenic state. Although data on their combined application are still limited, the preliminary results suggest that such an approach may improve the identification of high-risk patients and enable a more accurate prediction of adverse cardiovascular outcomes.

A cross-sectional study examining the relationship between serum Hcy and TnI in 194 consecutive patients with AMI showed a statistically significant correlation between these two biomarkers (r = 0.273, *p* < 0.001). Patients with moderate HHcy (≥15 μmol/L) exhibited significantly higher mean TnI concentrations compared to those with normal Hcy levels (18.4 ± 6.5 vs. 8.9 ± 8.6 ng/mL, *p* < 0.05). Multivariate logistic regression analysis revealed that subjects with moderate HHcy had a 7.09 times higher probability of elevated TnI concentrations, independent of other cardiovascular risk factors, indicating an association between Hcy and a more pronounced degree of myocardial damage. However, the authors acknowledged that the limited sample size and single-center design may restrict the generalizability of these findings [174]. Similar results were obtained in a case–control study by Kumar et al., which included 100 MI patients and 72 healthy controls. In this study, elevated Hcy concentrations were recorded in 98% of patients with positive TnT values compared to 18.06% of controls, with significantly higher mean Hcy levels in the TnT positive group. Univariate logistic regression revealed that a 0.1-unit increase in Hcy was associated with a 12.60% increased risk of TnT positivity (*p* < 0.0001). Interestingly, in the same study, no significant correlation of Hcy with other cardiac markers, such as CK-MB and LDH, was established, further emphasizing the specific relationship between Hcy and TnT [175]. This connection was also confirmed in a recent case–control study involving 80 patients with ACS, 40 patients with chronic stable angina, and 60 healthy controls. Both patient groups demonstrated significantly higher values of Hcy and troponin compared to healthy subjects, independent of sex. Interestingly, the study found no statistically significant differences in biomarker levels between diabetic patients and controls, which contrasts with expected metabolic influences. However, hypertension was identified as a substantial factor affecting cardiac marker levels in both acute and chronic coronary presentations [176]. A pioneering study examining the relationship between the degree of myocardial damage in ACS and plasma Hcy concentration demonstrated a pronounced association between these two parameters. This prospective study of 390 consecutive patients (205 with acute MI and 185 with unstable angina pectoris) demonstrated a statistically significant gradual increase in TnT values in accordance with increasing Hcy concentrations, with the highest cTn values registered in the group with the highest Hcy levels. A similar gradient was observed in patients with unstable angina pectoris. It is important to emphasize that in multivariate analysis, after adjustment for potential confounders (age, sex, final diagnosis, and applied therapy), the relationship between elevated Hcy concentrations and cTn remained statistically highly significant [177]. Accordingly, in addition to correlation with cTn, elevated serum Hcy levels have been shown to be associated with reduced ejection fraction, with both parameters independently reflecting the degree of myocardial damage in the context of ACS [178]. These findings indicate that elevated Hcy values are associated with an increased risk of ischemic myocardial damage, further confirming the potential value of simultaneous determination of both biomarkers in assessing the severity of ACS.

A study conducted on an asymptomatic population from the general community showed for the first time that Hcy levels are positively associated with the presence of detectable hs-cTn values, independent of age, sex, and other vascular risk factors. This large community-based cross-sectional study of 1497 asymptomatic subjects demonstrated an independent association between Hcy and subclinical myocardial damage. It is particularly significant that in a predefined subgroup analysis, this association was more pronounced in older participants, while it was not present in individuals younger than 65 years [179]. These findings suggest that the combined determination of hs-cTn and Hcy may have special value in the early detection of cardiovascular risk in elderly populations, enabling the timely implementation of preventive measures before the appearance of clinical manifestations of the disease. On the other hand, the prognostic value of the combined determination of Hcy and hs-cTn has also been demonstrated in the pediatric population. A study that included 80 pediatric patients with acute HF (age range 2 months to 6 years) and 80 matched controls showed that both biomarkers were significantly elevated in the acute phase of the disease, with their level of increase correlating with the severity of HF according to the Ross classification. Both values showed a significant correlation with echocardiographic parameters, including a positive correlation with cardiomegaly and a negative correlation with left ventricular ejection fraction. It is particularly significant that the simultaneous increase in both biomarkers was associated with unfavorable outcomes, with a mortality rate of 10% during a three-month follow-up [180].

Although a consistent positive association between Hcy and troponins has been documented in ACS and subclinical myocardial injury, the findings from patients with impaired renal function highlight a more complex and partly conflicting relationship. Meta-analysis demonstrated that elevated cardiac troponin levels are generally associated with increased mortality in chronic kidney disease (CKD) patients. However, significant heterogeneity exists, with larger population studies and those including younger patients showing no significant association between troponin and mortality outcomes [181]. Concurrently, Hcy levels did not significantly differ between CKD patients, hypertensive individuals, and healthy controls, suggesting that renal impairment does not uniformly drive Hcy elevation, nor does it provide a reliable stratification of cardiovascular risk in this setting [182]. These discordant patterns between biomarkers in CKD populations suggest that their established synergy may be population dependent, with potential limitations in specific disease states where conventional cardiovascular risk assessment frameworks may not apply.

Despite the limited number of studies directly investigating the synergistic use of hs-cTn and Hcy, the presented results indicate significant potential for such a multi-marker approach. The complementary nature of these biomarkers provides a more comprehensive insight into the complex pathophysiological processes that contribute to CVD. The demonstrated association between elevated Hcy concentrations and more pronounced myocardial damage quantified by cTn, as well as their joint prognostic value in different populations, from asymptomatic adults to pediatric patients with HF, suggests that the integration of both biomarkers could significantly improve cardiovascular risk stratification. Current evidence encompasses studies with varying sample sizes across diverse designs, although heterogeneity in methodological approaches and population characteristics limits the direct comparability of findings. However, the lack of large prospective studies directly evaluating the added value of their combined application necessitates further research to define optimal clinical algorithms, cut-off values, and the specific populations that would benefit most from this approach.

## 6. Challenges and Limitations in the Use of High-Sensitivity Troponins and Hcy

Despite significant diagnostic and prognostic potential, the interpretation of hs-cTn and Hcy concentrations in the clinical setting may be complicated by the specific limitations associated with each of these biomarkers. Understanding these factors is crucial for proper cardiovascular risk assessment and therapeutic decision-making.

### 6.1. Analytical and Pre-Analytical Limitations

Despite the exceptional clinical value of hs-cTn tests, their reliability can be limited by analytical variability, particularly the intermittent bias arising from changes in calibrators or reagents. Such analytical variations may significantly influence the allocation of patients across risk categories within accelerated diagnostic protocols, potentially leading to misclassification through false rule-out and rule-in determinations for NSTEMI. The problem is particularly pronounced when using absolute thresholds in rule-out algorithms, where patients presenting more than 2 to 3 h after symptom onset may be eligible for early discharge if hs-cTn concentrations fall below the limit of detection. Additionally, when combined pre-analytical uncertainty and coefficient of variation are elevated, erroneous delta values may occur for individual patients, resulting in incorrect follow-up strategies and therapeutic approaches. These analytical challenges underscore the complexity of maintaining objective acceptable performance in clinical practice, where emergency physicians typically accept a miss-rate for NSTEMI for less than 1%. These findings emphasize the need for rigorous monitoring of hs-cTn method stability in routine clinical practice, and potentially incorporating cTn values into multifactorial risk models that are less sensitive to analytical fluctuations [183].

Several technological advances offer potential solutions for these analytical challenges. Point-of-care testing (POCT) methods with high analytical sensitivity can significantly reduce turnaround times and enable diagnosis in diverse clinical settings, including ambulance, outpatient clinics, and home care environments, as these assays eliminate the need for sample centrifugation or complex preanalytical processing. The IFCC Committee provides standardized requirements for high-sensitivity assays and regularly updates analytical performance criteria. Future innovations include wearable devices capable of non-invasive transdermal monitoring and the integration of machine learning capabilities into POCT platforms to enhance accuracy while maintaining portability [184].

Clinical validation demonstrates the practical implementation of these solutions. The SPINCHIP hs-cTnI POCT achieved diagnostic performance equivalent to central laboratory assays while enabling rapid testing across multiple sample types, including capillary finger prick samples. The assay specific 0/1-h algorithm achieved 100% sensitivity for MI rule-out, demonstrating how POCT technology can maintain analytical precision while expanding diagnostic accessibility beyond traditional hospital laboratories [185].

Unlike hs-cTn, where analytical variations primarily impact clinical interpretation, for Hcy, challenges arise from both the pre-analytical sample processing phase and the analytical variability of determination methods. In the pre-analytical phase, EDTA-plasma is most commonly used, but if plasma is not separated from blood cells within 30 min, Hcy concentration can increase by up to 10% per hour. This is particularly significant given that the boundary between normal and mildly elevated values lies in a relatively narrow range (5–15 µmol/L), increasing the risk of misclassification of vascular risk—especially when sampling outside hospital facilities, where timely sample processing is often not feasible. Additionally, although different methods for determining Hcy provide comparable results, variability among methods and laboratories remains significant. Based on known biological variability, the ideal systematic measurement error should be less than 10%, and imprecision not higher than 5%; however, many available tests do not meet these criteria. The lack of test standardization and the absence of certified reference materials further complicate the reliable interpretation of results in clinical practice. Therefore, routine application of Hcy as a biomarker of vascular risk requires strictly controlled sample processing conditions and standardized analytical methods, which presents a significant challenge both in clinical studies and in everyday diagnostic practice [186,187].

### 6.2. Clinical Interpretation and Differential Diagnostics

hs-cTn has significantly improved the diagnosis of AMI, primarily due to their extremely high negative predictive value and shortening of the so-called “Tn blind” zone, enabling earlier AMI detection. These advantages have resulted in reduced Emergency Department stays and overall healthcare costs, while simultaneously recording a decrease, or at least no increase, in the use of non-invasive and invasive diagnostic procedures. Additionally, the introduction of more sensitive methods has enabled the recognition of milder forms of myocardial damage that would previously have remained undiagnosed, facilitating timely cardiovascular evaluation regardless of the etiology of the damage. However, the increased sensitivity of these tests brings certain diagnostic challenges, given that elevated hs-cTn concentrations are not specific to AMI. For example, the positive predictive value of the ESC “0/1 h” algorithm for confirming AMI is about 70%, implying that interpretation must be careful and in the context of the clinical picture and ECG findings. Although higher hs-cTn values more often indicate the presence of AMI, there is no clearly defined threshold value that would definitively differentiate this diagnosis. Theoretically, increased diagnostic sensitivity could lead to overdiagnosis, unnecessary interventions, and consequent hospitalization, but the available data indicate that such adverse effects are rarely recorded, especially when test implementation is conducted with adequate clinician education and application of standardized diagnostic protocols for patients with elevated hs-cTn but without evidence of MI [188,189].

An additional challenge in the clinical use of hs-cTn tests is the limited ability to differentiate between various causes of elevated cTn values. Although hs-cTn values are crucial for detecting myocardial damage, they are not sufficient by themselves to precisely distinguish Type 1 MI (which results from atherosclerotic plaque rupture and thrombosis) from other conditions that may also be accompanied by elevated cTn values, such as myocarditis, Takotsubo cardiomyopathy, pulmonary embolism with right-sided strain, severe hypertensive crisis, coronary vasospasm, or ischemia due to increased oxygen demand (so-called demand ischemia). This diagnostic non-specificity is particularly evident in emergency clinical situations when the speed and accuracy of decision-making are of crucial importance. Even the newest generations of hs-cTn tests do not allow reliable etiological classification based on a single measurement, since there is no universally accepted absolute threshold value that could reliably distinguish different forms of myocardial damage. Consequently, the diagnostic approach continues to depend on serial cTn measurements, interpreted within the context of the clinical presentation and ECG findings [190].

Unlike hs-cTn, whose role in diagnostic algorithms is clearly defined, Hcy as a marker of cardiovascular risk is characterized by significantly more complex clinical interpretation. Epidemiological studies consistently indicate an association between elevated Hcy concentrations and CVD, with this association further confirmed by experimental findings regarding its harmful effects on vascular homeostasis. However, a significant paradox is observed in clinical practice; interventional studies that used Hcy-lowering therapy, primarily based on B-vitamin and folate supplementation, did not show a proportional reduction in cardiovascular morbidity and mortality, despite the evident lowering of the Hcy values themselves. Findings from these studies range from neutral effects on CAD to inconclusive results in the context of cerebrovascular diseases [191,192,193,194].

This discrepancy between epidemiological correlations and limited intervention effects further complicates the clear positioning of Hcy in cardiac diagnostics. Diagnostic uncertainty is further amplified by the absence of universally accepted threshold values for risk stratification, as well as the presence of numerous confounding factors—including differences in methodology, variations in study design, and heterogeneity of examined populations. In light of these challenges, contemporary approaches increasingly treat Hcy as a complementary rather than a standalone biomarker in cardiovascular risk assessment [191].

### 6.3. Impact of Comorbidities and Physiological Factors

Numerous comorbidities, such as chronic renal insufficiency, HF, DM, and aging, can lead to chronic cTn elevation, further complicating the interpretation of hs-cTn test results. This phenomenon represents a particular diagnostic challenge in patients with suspected ACS, especially in older individuals in which borderline cTn values are often registered even in the absence of AIM. Studies indicate that hs-cTn tests have moderate incremental diagnostic value in patients with comorbidities, as well as in those admitted to the hospital several hours after symptom onset. It is important to emphasize that chronically elevated cTn values reflect actual myocardial damage and should not be interpreted as false positive findings. This further highlights the significance of dynamic monitoring of cTn concentrations as a key step in differentiating acute from chronic myocardial damage. Such an approach requires standardized diagnostic protocols and the continuous education of clinicians to avoid potentially unnecessary invasive procedures [195,196].

Hcy concentration is also subject to the influence of numerous factors, which complicates its interpretation in a clinical context. Age and sex are significant determinants, with males and older individuals showing higher values. Nutritional status, especially intake of folate and vitamins B6 and B12, directly affects Hcy levels. Lifestyle habits such as coffee consumption, smoking, and reduced physical activity are associated with elevated concentrations. Certain medications, including methotrexate, antiepileptics, and lipid-lowering drugs, interfere with Hcy metabolism. Renal function has a pronounced impact, with even moderate renal insufficiency potentially doubling Hcy values. Ethnic differences have also been documented, potentially linked to the genetic polymorphisms of enzymes involved in Hcy metabolism. These diverse influences significantly complicate the use of Hcy as a reliable biomarker of cardiovascular risk [197,198,199].

## 7. Future Directions: Advancing Cardiovascular Risk Prediction with Biomarkers

Despite significant progress in all areas of cardiovascular medicine, the importance of individualized early detection and precise diagnostics is increasingly recognized. Biomarkers play a key role in this process, but the currently available biomarkers face certain limitations, including insufficient specificity and pronounced interindividual variability [200]. hs-cTn and Hcy, despite their limitations, provide a solid foundation for the further development of diagnostic and prognostic tools in cardiology. Contemporary research is directed toward overcoming existing challenges through the development of new detection methods, personalized approaches to result interpretation, multi-marker strategies, and the integration of biomarkers into advanced risk assessment algorithms. These innovative approaches have the potential not only to improve the precision of cardiovascular risk stratification but also to transform prevention and therapeutic strategies in modern cardiology practice, with special significance for vulnerable populations and areas with limited resources.

### 7.1. Novel Methods of Detection

A significant advancement in cardiovascular diagnostics lies in the development of novel, less invasive, and more accessible methods for biomarker detection. Recent studies have demonstrated the feasibility of detecting high-sensitivity cardiac Tns (hs-cTn) in alternative biological fluids such as urine and saliva. In one study evaluating hs-cTn levels in first-morning urine samples from healthy individuals and patients with hypertension, significantly elevated cTn concentrations were observed in the hypertensive group. These findings highlight the potential for urine-based, non-invasive diagnostic assays, which due to their practicality, simplicity, and cost-effectiveness may serve as valuable tools not only for the monitoring of hypertension but also for broader cardiovascular risk assessment. This approach may be particularly advantageous in pediatric populations and in individuals for whom venipuncture is challenging, allowing for more frequent and less stressful monitoring of cardiovascular status [201]. Furthermore, emerging evidence has demonstrated a significant correlation between salivary and serum hs-cTnT levels in patients with AMI, supporting the feasibility of salivary-based testing. Such developments open new possibilities for simplified cardiovascular monitoring and even self-assessment by patients in outpatient or home-based settings [202,203].

In the field of Hcy detection, contemporary research is directed toward overcoming the limitations of traditional methods, which are often expensive, unavailable in resource-limited areas, and require complex laboratory equipment [187,204]. Notable advances have been made with the development of electrochemical detection techniques, particularly those employing screen-printed electrodes, which are characterized by simplicity, low cost, easier use, and portability. The sensitivity of these systems has been further enhanced by the incorporation of nanomaterials, such as gold nanoparticles, carbon nanotubes, and graphene [204]. A particularly promising innovation involves the use of aptamers (short fragments of DNA or RNA sequences) that specifically bind to Hcy, producing measurable changes in the electrochemical signal. Aptamer-based biosensors have demonstrated high specificity, reproducibility, and clinically acceptable accuracy in the analysis of biological samples. The development of such accessible and reliable tests could significantly advance Hcy monitoring and enable its wider application in cardiovascular risk assessment, especially in preventive medicine and screening of large populations [205].

In addition to electrochemical techniques and aptamer-based sensors, point-of-care tests represent a particularly promising direction in developing new approaches for Hcy detection. These tests, designed for use at the point of healthcare delivery without requiring complex laboratory infrastructure, could have a significant global impact in assessing folate deficiency. Hcy has proven to be a reliable functional biomarker for folate deficiency, with plasma levels inversely related to folate status. Compared to conventional biomarkers used to evaluate folate deficiency (serum folate and red blood cell folate), plasma Hcy offers several advantages, such as greater sample stability, simpler processing requirements, and more consistent reference values [206].

### 7.2. Personalized Interpretation of Threshold Values

Recent advances in the interpretation of cardiovascular risk biomarkers have increasingly shifted toward personalized approaches that account for individual patient characteristics, rather than relying on universal threshold values. The conventional use of fixed cut-off points for hs-cTn assays has demonstrated notable limitations, particularly in stratifying patients at intermediate risk, who represent a substantial proportion of those presenting with ACS [190].

Secondary analysis of the High-STEACS study demonstrated that a hs-cTnI concentration below 5 ng/L reliably identifies patients at very low risk for both immediate and future cardiac events, with a negative predictive value greater than 99.5%, independent of age, sex, and ECG findings. Applying this threshold allows for the identification of twice as many low-risk patients compared to using the assay’s detection limit alone. Conversely, hs-cTnI values between 5 ng/L and the diagnostic threshold are associated with a seven-fold increase in the risk of MI or death within one year. These results support the use of separate thresholds for stratification and diagnostics to improve risk assessment in patients with suspected ACS [207].

A recent analysis has shown that the use of hs-cTnI with threshold values of 2 ng/L and 6 ng/L enables the effective stratification of patients with stable ischemic heart disease into three risk categories for MACE: low-risk (<1% annual incidence), medium-risk (1–3% annually), and high-risk (>3% annually) cohorts. Patients with hs-cTnI values below 2 ng/L had a statistically significantly lower risk of MACE compared to the intermediate-risk group, whereas those with levels exceeding 6 ng/L exhibited a two-fold risk increase. Notably, among patients with undetectable hs-cTnI concentrations (10.7% of the cohort), no cardiovascular deaths were observed during the three-year follow-up period. Additionally, hs-cTnI was shown to enhance cardiovascular risk stratification beyond conventional atherosclerotic CVD models, enabling finer identification of patients who need more intensive or less aggressive therapy, including the potential use of lipid-lowering therapy [208].

As different hs-cTnT and hs-cTnI tests become widely accepted and implemented in clinical practice, the evaluation of data underlying the clinical threshold values (uniform and sex-specific) for each test becomes imperative. A lack of uniformity has been observed in defining “healthy” cohorts across different tests and studies, which has led to the development of biologically unequal clinical threshold values that can affect the diagnostic contribution of each hs-cTnT and hs-cTnI test. Of particular importance is the fact that applying uniform cTn threshold values for both sexes results in the systematic underdiagnosis of MI in women. Without prospective studies, it is impossible to assess the extent to which this underdiagnosis contributes to inequalities in the management and treatment of MI [209]. These findings emphasize the necessity of developing and implementing sex-specific algorithms for interpreting hs-cTn, which could significantly improve the precision of diagnostics and risk stratification in the female population.

These personalized approaches are increasingly being enhanced through machine-learning algorithms that can integrate multiple patient-specific factors simultaneously. The ARTEMIS diagnostic model demonstrates this advancement by utilizing data from over 27,000 patients across four continents to provide individualized MI risk probabilities rather than fixed thresholds [210]. The model incorporates eleven clinical variables, including sex, age, cardiovascular risk factors, and ECG findings, while accommodating multiple hs-cTn assays without requiring assay-specific cut-off values. Clinical validation in geographically distinct cohorts showed superior performance compared to guideline pathways, enabling a direct rule-out in 30–49% of patients versus 13–15% using conventional algorithms, while maintaining negative predictive values exceeding 99.6% [211].

A personalized approach to interpreting Hcy as a biomarker of cardiovascular risk is increasingly gaining importance. Contemporary research indicates pronounced gender differences in Hcy concentrations, with men consistently having higher values in all age groups compared to women, and this pattern persists during aging [212,213]. Additionally, aging itself represents a significant factor in increasing Hcy concentration, with the highest values recorded in individuals older than 60 years, independent of sex. Multiple mechanisms contribute to these changes in the elderly population, including slowed digestion and reduced absorption of vitamins necessary for Hcy metabolism, decreased activity of key enzymes involved in its metabolism, as well as a progressive decline in liver and kidney function [212]. These findings emphasize the need for introducing age- and sex-specific reference values for Hcy, to improve the precision of cardiovascular risk assessment and optimize clinical decision-making.

In light of these discoveries, the development of integrated algorithms that would automatically adjust Hcy interpretation to individual patient characteristics represents a significant step toward more precise cardiovascular risk stratification. Such personalized approaches could overcome the limitations of traditional fixed threshold values, enabling a more comprehensive assessment that takes into account the complex interaction of demographic, genetic, and metabolic factors [214]. However, for the broad implementation of personalized algorithms in clinical practice, their validation in large, prospective, multicenter studies including diverse patient populations and clinical scenarios is necessary. Such studies could define optimal approaches to personalizing Hcy threshold values and confirm their real contribution to improving diagnostic and prognostic accuracy in cardiovascular risk assessment.

Contemporary advances in personalized Hcy interpretation extend beyond demographic adjustments to encompass comprehensive 3P medicine approaches (predictive, personalized, and preventive). These strategies integrate genetic polymorphism affecting Hcy metabolism, particularly MTHFR variants, with individualized nutritional assessment and targeted interventions. Current management protocols now incorporate specific vitamin B6, B12, and folate supplementation regimens tailored to individual metabolic profiles, alongside specific lifestyle modifications and dietary strategies designed to maintain plasma Hcy concentrations below optimal thresholds. The development of integrated algorithms that automatically adjust interpretation based on genetic, nutritional, and clinical factors represents a next step toward precision cardiovascular risk assessment [215,216].

### 7.3. Integration of Multiple Biomarkers

The future development of cardiovascular medicine is increasingly directed toward the application of integrated biomarker panels that simultaneously reflect multiple pathological processes. This strategy aims to overcome the limitations of individual biomarkers by combining markers of myocardial injury, vascular dysfunction, inflammation, and hemodynamic stress to provide a more comprehensive and individualized assessment of cardiovascular risk.

Recent research indicates the particular importance of a multi-marker approach in specific populations with elevated cardiovascular risk. Patients with type 2 DM represent a group with a high prevalence of often asymptomatic CVD and a significant risk of vascular complications. However, individual risk can vary considerably, and current assessment methods have certain limitations. NT-proBNP and hs-cTnT, both individually and combined, independently predict incident HF and total CVD events in individuals with and without prediabetes and DM, providing incremental prognostic value beyond traditional risk factors. The highest risk occurs when both biomarkers are elevated, supporting the clinical utility of a multi-marker approach for cardiovascular risk stratification in a dysglycemic population [217].

A new perspective in the multi-marker approach involves integrating biomarkers of different pathophysiological mechanisms, especially in risk assessment for patients with HF. The combination of hs-cTn with natriuretic peptides (BNP and NT-proBNP) along with emerging markers, such as ST2, enables a more comprehensive evaluation of various pathophysiological pathways of HF. The Barcelona Bio-Heart Failure Risk Calculator, an innovative clinical algorithm that integrates hs-cTnT with NT-proBNP and ST2, has shown exceptional precision in predicting total mortality, hospitalization, and combined adverse events in these patients. This approach overcomes the limitations of individual biomarkers, even those traditionally considered the gold standard, and represents a significant step toward personalized risk assessment [218].

The synergistic effect of combining different biomarkers was demonstrated in a study that evaluated hs-CRP, Hcy, and fibrinogen in assessing the ten-year risk of CAD. The results clearly show a gradual increase in the ten-year risk of CAD with increasing circulating levels of the mentioned biomarkers. Statistical analyses showed that subjects with hs-CRP and Hcy values in the highest quartile have a significantly higher odds ratio (OR) for high risk of CAD (ten-year risk ≥ 20%), while such an association was not established for fibrinogen. These associations maintained statistical significance even after adjustment for traditional risk factors, including LDL cholesterol, triglycerides, body mass index, and diastolic blood pressure [219]. Similarly, Wang et al. determined that the combination of Hcy and hs-CRP can predict the development of arterial stiffness. Subjects with the highest levels of both biomarkers (Hcy ≥ 15.50 μmol/L and hs-CRP ≥ 0.82 μmol/L) had a 12.68 times higher risk of developing arterial stiffness during a 12-year follow-up compared to subjects with lower levels of Hcy and hs-CRP (Hcy ≤ 9.91 μmol/L and hs-CRP ≤ 0.19 μmol/L). This result remained statistically significant even after adjustment for potential confounding factors, indicating synergy between Hcy and hs-CRP in predicting cardiovascular changes, such as arterial stiffness [220].

Experimental evidence supports a mechanistic link between hs-cTn and Hcy. Notably, in comparative analyses across various cardiometabolic models in rats, a concomitant elevation of tHcy and hs-cTn was observed exclusively in the isoprenaline-induced MI model, thereby reinforcing both the biological plausibility and the potential clinical utility of their combined assessment [221].

Collectively, these findings reflect the growing trend in cardiovascular medicine toward the development and clinical implementation of sophisticated multi-marker panels that integrate biomarkers reflecting different pathophysiological processes. A combined approach that includes hs-cTn and Hcy as complementary markers of myocardial damage and vascular dysfunction, together with inflammatory and metabolic biomarkers, can potentially improve the precision of cardiovascular risk stratification. This approach is particularly valuable in identifying high-risk patients who might benefit most from preventive interventions, as well as in personalizing therapeutic strategies based on individual biomarker profiles.

Future investigations should focus on identifying the most informative biomarker combinations and on establishing standardized interpretative algorithms to enhance the clinical applicability of multi-marker strategies.

## 8. Conclusions

The integration of hs-cTn and Hcy into cardiovascular diagnostics and risk assessment reflects a paradigm shift toward more precise, personalized medicine. hs-cTn assays have substantially improved early detection of ACS and myocardial injury, offering high diagnostic accuracy and prognostic value across a wide spectrum of both cardiovascular and non-cardiovascular conditions. Meanwhile, elevated Hcy levels, despite being a well-established risk factor for vascular dysfunction and atherothrombosis, continue to pose interpretative challenges due to their susceptibility to multiple confounding factors and inconsistent responses to therapeutic interventions. A significant issue that continues to require clarification is the extent to which Hcy directly contributes to the development of vascular disease, as opposed to serving merely as a biomarker that indicates other metabolic changes affecting vascular function.

Nevertheless, a growing body of evidence suggests that the simultaneous use of hs-cTn and Hcy may offer complementary insights, capturing both myocardial damage and endothelial dysfunction. The combined use of hs-cTn and Hcy may offer added prognostic value, particularly in clinical scenarios where standard risk models fail to account for residual or complex cardiovascular risk. While preliminary studies support the utility of this combined strategy in various clinical settings, including ACS, HF, and even asymptomatic populations, large-scale prospective research is required to establish definitive thresholds, clarify temporal dynamics, and determine the additive value in routine clinical workflows. Future diagnostic frameworks should aim to incorporate such multi-marker models to enable timely, cost-effective, and population-specific cardiovascular risk management.

## Data Availability

This study is based on a narrative review of previously published literature. No new data were generated or analyzed, and data sharing is not applicable to this article.

## References

[1] Danese E., Montagnana M. (2016). An historical approach to the diagnostic biomarkers of acute coronary syndrome. Ann. Transl. Med..

[2] LaDue J.S., Wróblewski F., Karmen A. (1954). Serum Glutamic Oxaloacetic Transaminase Activity in Human Acute Transmural Myocardial Infarction. Science.

[3] Karmen A. (1955). A note on the spectrometric assay of glutamic-oxalacetic transaminase in human blood serum. J. Clin. Investig..

[4] Henry R.J., Chiamori N., Golub O.J., Berkman S. (1960). Revised Spectrophotometric Methods for the Determination of Glutamic-Oxalacetic Transaminase, Glutamic-Pyruvic Transaminase, and Lactic Acid Dehydrogenase. Am. J. Clin. Pathol..

[5] Hill B.R., Levi C. (1954). Elevation of a serum component in neoplastic disease. Cancer Res..

[6] Wróblewski F., Ruegsegger P., LaDue J.S. (1956). Serum Lactic Dehydrogenase Activity in Acute Transmural Myocardial Infarction. Science.

[7] Dreyfus J., Schapira G., Resnais J., Scebat L. (1960). Serum creatine kinase in the diagnosis of myocardial infarct. Rev. Fr. D’Etudes Clin. Biol..

[8] Roe C.R., Limbird L.E., Wagner G.S., Nerenberg S.T. (1972). Combined isoenzyme analysis in the diagnosis of myocardial injury: Application of electrophoretic methods for the detection and quantitation of the creatine phosphokinase MB isoenzyme. J. Lab. Clin. Med..

[9] (1979). Nomenclature and criteria for diagnosis of ischemic heart disease. Report of the Joint International Society and Federation of Cardiology/World Health Organization task force on standardization of clinical nomenclature. Circulation.

[10] Ebashi S. (1960). Calcium Binding and Relaxation in the Actomyosin System. J. Biochem..

[11] Ebashi S., Endo M., Otsuki I. (1969). Control of muscle contraction. Q. Rev. Biophys..

[12] Greaser M.L., Gergely J. (1971). Reconstitution of Tn Activity from Three Protein Components. J. Biol. Chem..

[13] Chong S.M., Jin J.-P.P. (2009). To Investigate Protein Evolution by Detecting Suppressed Epitope Structures. J. Mol. Evol..

[14] Sheng J.-J., Jin J.-P. (2014). Gene regulation, alternative splicing, and posttranslational modification of Tn subunits in cardiac development and adaptation: A focused review. Front. Physiol..

[15] Brotto M.A., Biesiadecki B.J., Brotto L.S., Nosek T.M., Jin J.-P. (2006). Coupled expression of Tn T and Tn I isoforms in single skeletal muscle fibers correlates with contractility. Am. J. Physiol. Physiol..

[16] Rasmussen M., Jin J.-P. (2021). Tn Variants as Markers of Skeletal Muscle Health and Diseases. Front. Physiol..

[17] Leite L., Matos P., Leon-Justel A., Espírito-Santo C., Rodríguez-Padial L., Rodrigues F., Orozco D., Redon J. (2022). High sensitivity Tns: A potential biomarkers of cardiovascular risk for primary prevention. Front. Cardiovasc. Med..

[18] Blanda V., Bracale U.M., Di Taranto M.D., Fortunato G. (2020). Galectin-3 in Cardiovascular Diseases. Int. J. Mol. Sci..

[19] Merino-Merino A., Gonzalez-Bernal J., Fernandez-Zoppino D., Saez-Maleta R., Perez-Rivera J.-A. (2021). The Role of Galectin-3 and ST2 in Cardiology: A Short Review. Biomolecules.

[20] Bai J., Han L., Liu H. (2020). Combined use of high-sensitivity ST2 and NT-proBNP for predicting major adverse cardiovascular events in coronary heart failure. Ann. Palliat. Med..

[21] Lee D.J., Aw T.-C. (2023). Natriuretic Peptides in Clinical Practice: A Current Review. J. Immunol. Sci..

[22] Fu Y., Wang X., Kong W. (2018). Hyperhomocysteinaemia and vascular injury: Advances in mechanisms and drug targets. Br. J. Pharmacol..

[23] Cao X., Wang T., Mu G., Chen Y., Xiang B., Zhu J., Shen Z. (2025). Dysregulated homocysteine metabolism and cardiovascular disease and clinical treatments. Mol. Cell. Biochem..

[24] Wu D.-F., Yin R.-X., Deng J.-L. (2024). Homocysteine, hyperhomocysteinemia, and H-type hypertension. Eur. J. Prev. Cardiol..

[25] McCully K.S. (1969). Vascular pathology of homocysteinemia: Implications for the pathogenesis of arteriosclerosis. Am. J. Pathol..

[26] Wilkinson J.M. (1980). Tn C from rabbit slow skeletal and cardiac muscle is the product of a single gene. Eur. J. Biochem..

[27] Li M.X., Hwang P.M. (2015). Structure and function of cardiac Tn C (TNNC1): Implications for heart failure, cardiomyopathies, and Tn modulating drugs. Gene.

[28] Van Eerd J.P., Takahashi K. (1976). Determination of the complete amino acid sequence of bovine cardiac Tn C. Biochemistry.

[29] Putkey J.A., Liu W., Sweeney H.L. (1991). Function of the N-terminal calcium-binding sites in cardiac/slow Tn C assessed in fast skeletal muscle fibers. J. Biol. Chem..

[30] McDonald K.S., Field L.J., Parmacek M.S., Soonpaa M., Leiden J.M., Moss R.L. (1995). Length dependence of Ca^2+^ sensitivity of tension in mouse cardiac myocytes expressing skeletal Tn C. J. Physiol..

[31] Fuchs F., Wang Y.P. (1991). Force, length, and Ca^2+^-Tn C affinity in skeletal muscle. Am. J. Physiol. Physiol..

[32] Hastings K.E. (1997). Molecular evolution of the vertebrate Tn I gene family. Cell Struct. Funct..

[33] Mullen A.J., Barton P.J. (2000). Structural characterization of the human fast skeletal muscle Tn I gene (TNNI2). Gene.

[34] Sheng J.-J., Jin J.-P. (2016). TNNI1, TNNI2 and TNNI3: Evolution, regulation, and protein structure-function relationships. Gene.

[35] de Tombe P.P., Belus A., Piroddi N., Scellini B., Walker J.S., Martin A.F., Tesi C., Poggesi C. (2007). Myofilament calcium sensitivity does not affect cross-bridge activation-relaxation kinetics. Am. J. Physiol. Regul. Integr. Comp. Physiol..

[36] Westfall M.V., Rust E.M., Metzger J.M. (1997). Slow skeletal Tn I gene transfer, expression, and myofilament incorporation enhances adult cardiac myocyte contractile function. Proc. Natl. Acad. Sci. USA.

[37] Hunkeler N.M., Kullman J., Murphy A.M. (1991). Tn I isoform expression in human heart. Circ. Res..

[38] Wei B., Jin J.-P. (2011). Tn T isoforms and posttranscriptional modifications: Evolution, regulation and function. Arch. Biochem. Biophys..

[39] Wei B., Jin J.-P. (2016). TNNT1, TNNT2, and TNNT3: Isoform genes, regulation, and structure-function relationships. Gene.

[40] Jin J.-P., Zhang Z., Bautista J.A. (2008). Isoform diversity, regulation, and functional adaptation of Tn and calponin. Crit. Rev. Eukaryot. Gene Expr..

[41] Farmakis D., Richter D., Chronopoulou G., Goumas G., Kountouras D., Mastorakou A., Papingiotis G., Hahalis G., Tsioufis K. (2024). High-sensitivity cardiac Tn I for cardiovascular risk stratification in apparently healthy individuals. Hell. J. Cardiol..

[42] Apple F.S., Ler R., Murakami M.M. (2012). Determination of 19 Cardiac Tn I and T Assay 99th Percentile Values from a Common Presumably Healthy Population. Clin. Chem..

[43] Neumann J.T., Twerenbold R., Ojeda F., Sörensen N.A., Chapman A.R., Shah A.S., Anand A., Boeddinghaus J., Nestelberger T., Badertscher P. (2019). Application of High-Sensitivity Tn in Suspected Myocardial Infarction. N. Engl. J. Med..

[44] Collet J.-P., Thiele H., Barbato E., Barthélémy O., Bauersachs J., Bhatt D.L., Dendale P., Dorobantu M., Edvardsen T., Folliguet T. (2021). 2020 ESC Guidelines for the management of acute coronary syndromes in patients presenting without persistent ST-segment elevation. Eur. Heart J..

[45] Sandoval Y., Januzzi J.L., Jaffe A.S. (2020). Cardiac Tn for Assessment of Myocardial Injury in COVID-19: JACC Review Topic of the Week. J. Am. Coll. Cardiol..

[46] Farmakis D., Mueller C., Apple F.S. (2020). High-sensitivity cardiac Tn assays for cardiovascular risk stratification in the general population. Eur. Heart J..

[47] Lippi G., Cervellin G. (2012). Degradation of Tn i in serum or plasma: Mechanisms, and analytical and clinical implications. Semin. Thromb. Hemost..

[48] Thygesen K., Alpert J.S., Jaffe A.S., Chaitman B.R., Bax J.J., Morrow D.A., White H.D. (2018). Fourth Universal Definition of Myocardial Infarction (2018). Glob. Heart.

[49] Sanfelice D., Sanz-Hernández M., de Simone A., Bullard B., Pastore A. (2016). Toward Understanding the Molecular Bases of Stretch Activation: A structural comparison of the two Tn c isoforms of lethocerus. J. Biol. Chem..

[50] Aimo A., Gaggin H.K., Barison A., Emdin M., Januzzi J.L.J. (2019). Imaging, Biomarker, and Clinical Predictors of Cardiac Remodeling in Heart Failure With Reduced Ejection Fraction. JACC Heart Fail..

[51] Gafane-Matemane L.F., Mokae N.L., Breet Y., Malan L. (2020). Relation of the renin-angiotensin-aldosterone system with potential cardiac injury and remodelling: The SABPA study. Blood Press..

[52] Lyngbakken M.N., Vigen T., Ihle-Hansen H., Brynildsen J., Berge T., Rønning O.M., Tveit A., Røsjø H., Omland T. (2021). Cardiac Tn I measured with a very high sensitivity assay predicts subclinical carotid atherosclerosis: The Akershus Cardiac Examination 1950 Study. Clin. Biochem..

[53] Wang W., Schulze C.J., Suarez-Pinzon W.L., Dyck J.R.B., Sawicki G., Schulz R. (2002). Intracellular action of matrix metalloproteinase-2 accounts for acute myocardial ischemia and reperfusion injury. Circulation.

[54] Chapman A.R., Adamson P.D., Mills N.L. (2017). Assessment and classification of patients with myocardial injury and infarction in clinical practice. Heart.

[55] Hessel M.H.M., Atsma D.E., van der Valk E.J.M., Bax W.H., Schalij M.J., van der Laarse A. (2008). Release of cardiac Tn I from viable cardiomyocytes is mediated by integrin stimulation. Pflug. Arch..

[56] Sabatine M.S., Morrow D.A., De Lemos J.A., Omland T., Desai M.Y., Tanasijevic M., Hall C., McCabe C.H., Braunwald E. (2004). Acute changes in circulating natriuretic peptide levels in relation to myocardial ischemia. J. Am. Coll. Cardiol..

[57] Canty J.M.J. (2022). Myocardial injury, Tn release, and cardiomyocyte death in brief ischemia, failure, and ventricular remodeling. Am. J. Physiol. Heart Circ. Physiol..

[58] Chaulin A.M. (2022). The Metabolic Pathway of Cardiac Tns Release: Mechanisms and Diagnostic Role. Cardiol. Res..

[59] Mair J. (2025). The Pathophysiology of Cardiac Troponin Release and the Various Circulating Cardiac Troponin Forms—Potential Clinical Implications. J. Clin. Med..

[60] Bergmann O., Bhardwaj R.D., Bernard S., Zdunek S., Barnabé-Heider F., Walsh S., Zupicich J., Alkass K., Buchholz B.A., Druid H. (2009). Evidence for cardiomyocyte renewal in humans. Science.

[61] Rovira M., Borràs D.M., Marques I.J., Puig C., Planas J. (2018). V Physiological Responses to Swimming-Induced Exercise in the Adult Zebrafish Regenerating Heart. Front. Physiol..

[62] Giacca M. (2020). Cardiac Regeneration After Myocardial Infarction: An Approachable Goal. Curr. Cardiol. Rep..

[63] Li L., Hessel M., van der Valk L., Bax M., van der Linden I., van der Laarse A. (2004). Partial and delayed release of Tn-I compared with the release of lactate dehydrogenase from necrotic cardiomyocytes. Pflug. Arch..

[64] Schwartz P., Piper H.M., Spahr R., Spieckermann P.G. (1984). Ultrastructure of cultured adult myocardial cells during anoxia and reoxygenation. Am. J. Pathol..

[65] Walter S., Carlsson J., Schröder R., Neuhaus K.L., Sorges E., Tebbe U. (1999). Enzymatic markers of reperfusion in acute myocardial infarct. With data from the ISAM study. Herz.

[66] Christenson R.H., Newby L.K., Ohman E.M. (1997). Cardiac markers in the assessment of acute coronary syndromes. Md. Med. J..

[67] Cummins B., Auckland M.L., Cummins P. (1987). Cardiac-specific Tn-I radioimmunoassay in the diagnosis of acute myocardial infarction. Am. Heart J..

[68] Larue C., Defacque-Lacquement H., Calzolari C., Le Nguyen D., Pau B. (1992). New monoclonal antibodies as probes for human cardiac Tn I: Epitopic analysis with synthetic peptides. Mol. Immunol..

[69] Bodor G.S., Porter S., Landt Y., Ladenson J.H. (1992). Development of monoclonal antibodies for an assay of cardiac Tn-I and preliminary results in suspected cases of myocardial infarction. Clin. Chem..

[70] Adams J.E., Bodor G.S., Dávila-Román V.G., Delmez J.A., Apple F.S., Ladenson J.H., Jaffe A.S. (1993). Cardiac Tn I. A marker with high specificity for cardiac injury. Circulation.

[71] Lippi G., Salvagno G.L., Da Rin G., Giavarina D. (2014). Harmonization of contemporary-sensitive Tn I immunoassays: Calibration may only be a part of the problem. Riv. Ital. Med. Lab..

[72] Salvagno G.L., Giavarina D., Meneghello M., Musa R., Aloe R., Da Rin G., Lippi G. (2014). Multicenter comparison of four contemporary sensitive Tn immunoassays. J. Med. Biochem..

[73] Katus H.A., Remppis A., Looser S., Hallermeier K., Scheffold T., Kübler W. (1989). Enzyme linked immuno assay of cardiac Tn T for the detection of acute myocardial infarction in patients. J. Mol. Cell. Cardiol..

[74] Katus H.A., Looser S., Hallermayer K., Remppis A., Scheffold T., Borgya A., Essig U., Geuss U. (1992). Development and in vitro characterization of a new immunoassay of cardiac Tn T. Clin. Chem..

[75] Müller-Bardorff M., Hallermayer K., Schröder A., Ebert C., Borgya A., Gerhardt W., Remppis A., Zehelein J., Katus H.A. (1997). Improved Tn T ELISA specific for cardiac Tn T isoform: Assay development and analytical and clinical validation. Clin. Chem..

[76] Hallermayer K., Klenner D., Vogel R. (1999). Use of recombinant human cardiac Tn T for standardization of third generation Tn T methods. Scand. J. Clin. Lab. Investig. Suppl..

[77] Hermsen D., Apple F., Garcia-Beltràn L., Jaffe A., Karon B., Lewandrowski E., Mühlbacher A., Müller R., Ordóñez J., Pagani F. (2007). Results from a multicenter evaluation of the 4th generation Elecsys Tn T assay. Clin. Lab..

[78] Giannitsis E., Kurz K., Hallermayer K., Jarausch J., Jaffe A.S., Katus H.A. (2010). Analytical validation of a high-sensitivity cardiac Tn T assay. Clin. Chem..

[79] Eggers K.M., Jernberg T., Lindahl B. (2017). Unstable Angina in the Era of Cardiac Tn Assays with Improved Sensitivity—A Clinical Dilemma. Am. J. Med..

[80] deFilippi C.R., de Lemos J.A., Christenson R.H., Gottdiener J.S., Kop W.J., Zhan M., Seliger S.L. (2010). Association of Serial Measures of Cardiac Tn T Using a Sensitive Assay With Incident Heart Failure and Cardiovascular Mortality in Older Adults. JAMA.

[81] Diez M., Talavera M.L., Conde D.G., Campos R., Acosta A., Trivi M.S. (2016). High-sensitivity Tn is associated with high risk clinical profile and outcome in acute heart failure. Cardiol. J..

[82] Raber I., McCarthy C.P., Januzzi J.L. (2021). A Test in Context: Interpretation of High-Sensitivity Cardiac Tn Assays in Different Clinical Settings. J. Am. Coll. Cardiol..

[83] Feng J., Schaus B.J., Fallavollita J.A., Lee T.C., Canty J.M.J. (2001). Preload induces Tn I degradation independently of myocardial ischemia. Circulation.

[84] Aimo A., Januzzi J.L.J., Vergaro G., Ripoli A., Latini R., Masson S., Magnoli M., Anand I.S., Cohn J.N., Tavazzi L. (2018). Prognostic Value of High-Sensitivity Tn T in Chronic Heart Failure: An Individual Patient Data Meta-Analysis. Circulation.

[85] Chin C.W.L., Shah A.S.V., McAllister D.A., Joanna Cowell S., Alam S., Langrish J.P., Strachan F.E., Hunter A.L., Maria Choy A., Lang C.C. (2014). High-sensitivity Tn I concentrations are a marker of an advanced hypertrophic response and adverse outcomes in patients with aortic stenosis. Eur. Heart J..

[86] Sandoval Y., Apple F.S., Mahler S.A., Body R., Collinson P.O., Jaffe A.S. (2022). High-Sensitivity Cardiac Tn and the 2021 AHA/ACC/ASE/CHEST/SAEM/SCCT/SCMR Guidelines for the Evaluation and Diagnosis of Acute Chest Pain. Circulation.

[87] Gulati M., Levy P.D., Mukherjee D., Amsterdam E., Bhatt D.L., Birtcher K.K., Blankstein R., Boyd J., Bullock-Palmer R.P., Conejo T. (2021). 2021 AHA/ACC/ASE/CHEST/SAEM/SCCT/SCMR Guideline for the Evaluation and Diagnosis of Chest Pain: A Report of the American College of Cardiology/American Heart Association Joint Committee on Clinical Practice Guidelines. Circulation.

[88] Jülicher P., Varounis C. (2022). Estimating the cost-effectiveness of screening a general population for cardiovascular risk with high-sensitivity Tn-I. Eur. Heart J.-Qual. Care Clin. Outcomes.

[89] Bajic Z., Sobot T., Skrbic R., Stojiljkovic M.P., Ponorac N., Matavulj A., Djuric D.M. (2022). Homocysteine, Vitamins B6 and Folic Acid in Experimental Models of Myocardial Infarction and Heart Failure—How Strong Is That Link?. Biomolecules.

[90] Škovierová H., Vidomanová E., Mahmood S., Sopková J., Drgová A., Červeňová T., Halašová E., Lehotský J. (2016). The molecular and cellular effect of homocysteine metabolism imbalance on human health. Int. J. Mol. Sci..

[91] De Matteis C., Crudele L., Di Buduo E., Cantatore S., Gadaleta R.M., Cariello M., Suppressa P., Antonica G., Berardi E., Graziano G. (2024). Hyperhomocysteinemia is linked to MASLD. Eur. J. Intern. Med..

[92] Stea T.H., Mansoor M.A., Wandel M., Uglem S., Frølich W. (2008). Changes in predictors and status of homocysteine in young male adults after a dietary intervention with vegetables, fruits and bread. Eur. J. Nutr..

[93] Ganguly P., Alam S.F. (2015). Role of homocysteine in the development of cardiovascular disease. Nutr. J..

[94] Kaye A.D., Jeha G.M., Pham A.D., Fuller M.C., Lerner Z.I., Sibley G.T., Cornett E.M., Urits I., Viswanath O., Kevil C.G. (2020). Folic Acid Supplementation in Patients with Elevated Homocysteine Levels. Adv. Ther..

[95] Roy S., Sable P., Khaire A., Randhir K., Kale A., Joshi S. (2014). Effect of maternal micronutrients (folic acid and vitamin B12) and omega 3 fatty acids on indices of brain oxidative stress in the offspring. Brain Dev..

[96] Vezzoli A., Dellanoce C., Caimi T.M., Vietti D., Montorsi M., Mrakic-Sposta S., Accinni R. (2020). Influence of dietary supplementation for hyperhomocysteinemia treatments. Nutrients.

[97] Zhang C.Y., Xu R.Q., Wang X.Q., Sun L.F., Mo P., Cai R.J., Lin X.Z., Luo C.F., Ou W.C., Lu L.J. (2023). Comprehensive transcriptomics and metabolomics analyses reveal that hyperhomocysteinemia is a high risk factor for coronary artery disease in a chinese obese population aged 40–65: A prospective cross-sectional study. Cardiovasc. Diabetol..

[98] Perła-Kaján J., Twardowski T., Jakubowski H. (2007). Mechanisms of homocysteine toxicity in humans. Amino Acids.

[99] Tyagi N., Sedoris K.C., Steed M., Ovechkin A.V., Moshal K.S., Tyagi S.C. (2005). Mechanisms of homocysteine-induced oxidative stress. Am. J. Physiol.-Heart Circ. Physiol..

[100] Lehotsky J., Petras M., Kovalska M., Tothova B., Drgova A., Kaplan P. (2015). Mechanisms Involved in the Ischemic Tolerance in Brain: Effect of the Homocysteine. Cell. Mol. Neurobiol..

[101] Xi H., Zhang Y., Xu Y., Yang W.Y., Jiang X., Sha X., Cheng X., Wang J., Qin X., Yu J. (2016). Caspase-1 Inflammasome Activation Mediates Homocysteine-Induced Pyrop-Apoptosis in Endothelial Cells. Circ. Res..

[102] Shi W., Zhang J., Zhao W., Yue M., Ma J., Zeng S., Tang J., Wang Y., Zhou Z. (2024). Intracellular Iron Deficiency and Abnormal Metabolism, Not Ferroptosis, Contributes to Homocysteine-Induced Vascular Endothelial Cell Death. Biomedicines.

[103] Valez V., Cassina A., Batinic-Haberle I., Kalyanaraman B., Ferrer-Sueta G., Radi R. (2013). Peroxynitrite formation in nitric oxide-exposed submitochondrial particles: Detection, oxidative damage and catalytic removal by Mn-porphyrins. Arch. Biochem. Biophys..

[104] Hu H., Wang C., Jin Y., Meng Q., Liu Q., Liu K., Sun H. (2016). Alpha-lipoic acid defends homocysteine-induced endoplasmic reticulum and oxidative stress in HAECs. Biomed. Pharmacother..

[105] Sibrian-Vazquez M., Escobedo J.O., Lim S., Samoei G.K., Strongin R.M. (2010). Homocystamides promote free-radical and oxidative damage to proteins. Proc. Natl. Acad. Sci. USA.

[106] Lai W.K.C., Kan M.Y. (2015). Homocysteine-induced endothelial dysfunction. Ann. Nutr. Metab..

[107] Chatterjee B., Fatima F., Seth S., Sinha Roy S. (2024). Moderate Elevation of Homocysteine Induces Endothelial Dysfunction through Adaptive UPR Activation and Metabolic Rewiring. Cells.

[108] Zhang Z.-Z., Yuan K., Yue H.-T., Yuan F.-H., Bi H.-T., Weng S.-P., He J.-G., Chen Y.-H. (2016). Identification and functional characterization of an endoplasmic reticulum oxidoreductin 1-$α$ gene in Litopenaeus vannamei. Dev. Comp. Immunol..

[109] Shai I., Stampfer M.J., Ma J., Manson J.E., Hankinson S.E., Cannuscio C., Selhub J., Curhan G., Rimm E.B. (2004). Homocysteine as a risk factor for coronary heart diseases and its association with inflammatory biomarkers, lipids and dietary factors. Atherosclerosis.

[110] Tian W., Ju J., Guan B., Wang T., Zhang J., Song L., Xu H. (2025). Role of hyperhomocysteinemia in atherosclerosis: From bench to bedside. Ann. Med..

[111] Yuan D., Chu J., Lin H., Zhu G., Qian J., Yu Y., Yao T., Ping F., Chen F., Liu X. (2023). Mechanism of homocysteine-mediated endothelial injury and its consequences for atherosclerosis. Front. Cardiovasc. Med..

[112] Williams K.T., Schalinske K.L. (2010). Homocysteine metabolism and its relation to health and disease. BioFactors.

[113] Bernardi M., Paneni F., Sabouret P. (2024). Homocysteine: A futile comeback or a promising tool for the risk assessment of hypertensive patients?. Eur. J. Prev. Cardiol..

[114] Qin X., Li Y., Sun N., Wang H., Zhang Y., Wang J., Li J., Xu X., Liang M., Nie J. (2017). Elevated homocysteine concentrations decrease the antihypertensive effect of angiotensin-converting enzyme inhibitors in hypertensive patients. Arterioscler. Thromb. Vasc. Biol..

[115] Calim A. (2020). The Relation between Homocysteine Levels in Patients with Acute Coronary Syndrome and Grace Score. SiSli Etfal Hastan. Tip Bul./Med. Bull. Sisli Hosp..

[116] Singh S.K., Jha R.K., Ambad R.S., Jha R.K. (2025). Comparative Analysis of Biomarkers in CAD: Evaluating Homocysteine, Lipid HS-CRP, Apo A, and ADMA. J. Pharm. Bioallied Sci..

[117] Niazi A.A., Karajibani M., Ghassami K., Montazerifar F., Iranneghad M., Bolouri A. (2021). A Comparison of Homocysteine, Tn, Cobalamin and Folate Status in Acute Myocardial Infarction Patients and Healthy Subjects. Adv. Hum. Biol..

[118] Spence J.D. (2024). Homocysteine and Myocardial Injury. JACC Asia.

[119] Ullah H., Huma S., Naeem L., Yasin G., Ashraf M., Tahir N., Yunus M., Shabana H., Shalaby A., Hassan Ali A.A. (2025). Correlation of Serum Homocysteine Levels With Various Types of Coronary Syndromes (CS) and In-Hospital Mortality—A Multicenter Study. Int. J. Gen. Med..

[120] Tan X., Tang F., Tian W., Zhang Y., Fang S., Yang S., Wang S., Yu B. (2024). Homocysteine Metabolism, Subclinical Myocardial Injury, and Cardiovascular Mortality in the General Population. JACC Asia.

[121] Rawashdeh S.I., Al-Mistarehi A.H., Yassin A., Rabab’ah W., Skaff H., Ibdah R. (2020). A concurrent ischemic stroke, myocardial infarction, and aortic thrombi in a young patient with hyperhomocysteinemia: A case report. Int. Med. Case Rep. J..

[122] Zheng X., Guo D., Peng H., Zhong C., Bu X., Xu T., Zhu Z., Wang A., Chen J., Xu T. (2019). Platelet counts affect the prognostic value of homocysteine in acute ischemic stroke patients. Atherosclerosis.

[123] Balmforth D., Harky A., Adams B., Yap J., Shipolini A., Roberts N., Uppal R., Bashir M. (2019). Is there a role for biomarkers in thoracic aortic aneurysm disease?. Gen. Thorac. Cardiovasc. Surg..

[124] Aday A.W., Duran E.K., Van Denburgh M., Kim E., Christen W.G., Manson J.E., Ridker P.M., Pradhan A.D. (2021). Homocysteine Is Associated with Future Venous Thromboembolism in 2 Prospective Cohorts of Women. Arterioscler. Thromb. Vasc. Biol..

[125] Manolescu B.N., Oprea E., Farcasanu I.C., Berteanu M., Cercasov C. (2010). Homocysteine and vitamin therapy in stroke prevention and treatment: A review. Acta Biochim. Pol..

[126] Xie W., Shan Y., Wu Z., Liu N., Yang J., Zhang H., Sun S., Chi J., Feng W., Lin H. (2023). Herpud1 deficiency alleviates homocysteine-induced aortic valve calcification. Cell Biol. Toxicol..

[127] Luo Z., Tang K., Huang G., Wang X., Zhou S., Dai D., Yang H., Jiang W. (2024). Homocysteine concentration in coronary artery disease and severity of coronary lesions. J. Cell. Mol. Med..

[128] Kim B.J., Seo M., Huh J.K., Kwon C.H., Kim J.T., Sung K.C., Kim B.S., Kang J.H. (2011). Associations of plasma homocysteine levels with arterial stiffness in prehypertensive individuals. Clin. Exp. Hypertens..

[129] Weiss N. (2005). Mechanisms of Increased Vascular Oxidant Stress in Hyperhomocysteinemia and Its Impact on Endothelial Function. Curr. Drug Metab..

[130] Wang G., Woo C.W.H., Sung F.L., Siow Y.L., Karmin O. (2002). Increased Monocyte Adhesion to Aortic Endothelium in Rats with Hyperhomocysteinemia: Role of Chemokine and Adhesion Molecules. Arterioscler. Thromb. Vasc. Biol..

[131] Ndrepepa G., Braun S., Schulz S., Mehilli J., Schömig A., Kastrati A. (2011). High-Sensitivity Troponin T Level and Angiographic Severity of Coronary Artery Disease. Am. J. Cardiol..

[132] Loscalzo J. (2001). Nitric Oxide Insufficiency, Platelet Activation, and Arterial Thrombosis. Circ. Res..

[133] Humphrey J.D. (2021). Mechanisms of Vascular Remodeling in Hypertension. Am. J. Hypertens..

[134] Zhang H., Liu Z., Ma S., Zhang H., Kong F., He Y., Yang X., Wang Y., Xu H., Yang A. (2016). Ratio of S-adenosylmethionine to S-adenosylhomocysteine as a sensitive indicator of atherosclerosis. Mol. Med. Rep..

[135] Yang A.N., Zhang H., Zhang H.P., Sun Y., Yang X.L., Wang N., Zhu G., Xu H., Ma S.C., Zhang Y. (2015). High-methionine diets accelerate atherosclerosis by HHcy-mediated FABP4 gene demethylation pathway via DNMT1 in ApoE-/- mice. FEBS Lett..

[136] Wang X., Chen Z., Tian W., Zhang J., Li Q., Ju J., Xu H., Chen K. (2023). Plasma homocysteine levels and risk of congestive heart failure or cardiomyopathy: A Mendelian randomization study. Front. Cardiovasc. Med..

[137] Di Cesare M., Perel P., Taylor S., Kabudula C., Bixby H., Gaziano T.A., McGhie D.V., Mwangi J., Pervan B., Narula J. (2024). The Heart of the World. Glob. Heart.

[138] Bhatnagar S., Jain M. (2024). Unveiling the Role of Biomarkers in Cardiovascular Risk Assessment and Prognosis. Cureus.

[139] Jigoranu R.A., Roca M., Costache A.-D., Mitu O., Oancea A.-F., Miftode R.-S., Haba M.Ș.C., Botnariu E.G., Maștaleru A., Gavril R.-S. (2023). Novel Biomarkers for Atherosclerotic Disease: Advances in Cardiovascular Risk Assessment. Life.

[140] Sabbatinelli J., Sbriscia M., Olivieri F., Giuliani A. (2024). Integrating cardiovascular risk biomarkers in the context of inflammaging. Aging.

[141] Tsoupras A., Lordan R., Zabetakis I. (2019). Cardiovascular Risk: Assumptions, Limitations, and Research. The Impact of Nutrition and Statins on Cardiovascular Diseases.

[142] Gigante B. (2023). Present and Future Perspectives on the Role of Biomarkers in Atherosclerotic Cardiovascular Disease Risk Stratification. Eur. Cardiol. Rev..

[143] Ananthan K., Lyon A.R. (2020). The Role of Biomarkers in Cardio-Oncology. J. Cardiovasc. Transl. Res.

[144] Sabkaewyod P., Vathesatogkit P., Sritara P. (2024). Clinical Significance of Undetectable High-sensitivity Cardiac Tn I in Thai Individuals with Low Cardiovascular Risk. J. Asian Pac. Soc. Cardiol..

[145] Unadkat S.V., Padhi B.K., Bhongir A.V., Gandhi A.P., Shamim M.A., Dahiya N., Satapathy P., Rustagi S., Khatib M.N., Gaidhane A. (2024). Association between homocysteine and coronary artery disease-trend over time and across the regions: A systematic review and meta-analysis. Egypt Heart J..

[146] Karger A.B., Nomura S.O., Guan W., Garg P.K., Tison G.H., Szklo M., Budoff M.J., Tsai M.Y. (2024). Association between elevated total homocysteine and heart failure risk in the Multi-Ethnic Study of Atherosclerosis cohort. J. Am. Heart Assoc..

[147] Zhang Z., Gu X., Fang X., Tang Z., Guan S., Liu H., Wu X., Wang C., Zhao Y. (2020). Homocysteine and the Risk of Cardiovascular Events and All-Cause Death in Elderly Population: A Community-Based Prospective Cohort Study. Ther. Clin. Risk Manag..

[148] Codoñer-Franch P., Alonso-Iglesias E., Patel V.B., Preedy V.R. (2016). Homocysteine as a Biomarker in Vascular Disease. Biomarkers in Cardiovascular Disease.

[149] Mendez D.Y., Zhou M., Brynedal B., Gudjonsdottir H., Tynelius P., Lagerros Y.T., Lager A. (2025). Risk stratification for cardiovascular disease: A comparative analysis of cluster analysis and traditional prediction models. Eur. J. Prev. Cardiol..

[150] Kim S.J., Mesquita F.C.P., Hochman-Mendez C. (2023). New Biomarkers for Cardiovascular Disease. Tex. Heart Inst. J..

[151] Willeit P., Welsh P., Evans J.D.W., Tschiderer L., Boachie C., Jukema J.W., Ford I., Trompet S., Stott D.J., Kearney P.M. (2017). High-Sensitivity Cardiac Tn Concentration and Risk of First-Ever Cardiovascular Outcomes in 154,052 Participants. J. Am. Coll. Cardiol..

[152] Katsioupa M., Kourampi I., Oikonomou E., Tsigkou V., Theofilis P., Charalambous G., Marinos G., Gialamas I., Zisimos K., Anastasiou A. (2023). Novel Biomarkers and Their Role in the Diagnosis and Prognosis of Acute Coronary Syndrome. Life.

[153] Nestelberger T., Boeddinghaus J., Wussler D., Twerenbold R., Badertscher P., Wildi K., Miró Ò., López B., Martin-Sanchez F.J., Muzyk P. (2019). Predicting Major Adverse Events in Patients With Acute Myocardial Infarction. J. Am. Coll. Cardiol..

[154] Mueller C., Giannitsis E., Christ M., Ordóñez-Llanos J., deFilippi C., McCord J., Body R., Panteghini M., Jernberg T., Plebani M. (2016). Multicenter Evaluation of a 0-Hour/1-Hour Algorithm in the Diagnosis of Myocardial Infarction With High-Sensitivity Cardiac Tn T. Ann. Emerg. Med..

[155] Paiva L., Vieira M.J., Baptista R., Ferreira M.J., Gonçalves L. (2024). Unstable Angina: Risk Stratification for Significant Coronary Artery Disease in The Era of High-Sensitivity Cardiac Tn. Glob. Heart.

[156] Yan I., Börschel C.S., Neumann J.T., Sprünker N.A., Makarova N., Kontto J., Kuulasmaa K., Salomaa V., Magnussen C., Iacoviello L. (2020). High-Sensitivity Cardiac Tn I Levels and Prediction of Heart Failure: Results From the BiomarCaRE Consortium. JACC Heart Fail..

[157] Evans J.D.W., Dobbin S.J.H., Pettit S.J., Di Angelantonio E., Willeit P. (2018). High-Sensitivity Cardiac Tn and New-Onset Heart Failure: A Systematic Review and Meta-Analysis of 67,063 Patients With 4165 Incident Heart Failure Events. JACC Heart Fail..

[158] Lyon A.R., López-Fernández T., Couch L.S., Asteggiano R., Aznar M.C., Bergler-Klein J., Boriani G., Cardinale D., Cordoba R., Cosyns B. (2022). 2022 ESC Guidelines on cardio-oncology developed in collaboration with the European Hematology Association (EHA), the European Society for Therapeutic Radiology and Oncology (ESTRO) and the International Cardio-Oncology Society (IC-OS). Eur. Heart J..

[159] Lv X., Pan C., Guo H., Chang J., Gao X., Wu X., Zhi X., Ren C., Chen Q., Jiang H. (2023). Early diagnostic value of high-sensitivity cardiac Tn T for cancer treatment-related cardiac dysfunction: A meta-analysis. ESC Heart Fail..

[160] Romann S.W., Finke D., Heckmann M.B., Hund H., Giannitsis E., Katus H.A., Frey N., Lehmann L.H. (2024). Cardiological parameters predict mortality and cardiotoxicity in oncological patients. ESC Heart Fail..

[161] Battault S., Renguet E., Van Steenbergen A., Horman S., Beauloye C., Bertrand L. (2020). Myocardial glucotoxicity: Mechanisms and potential therapeutic targets. Arch. Cardiovasc. Dis..

[162] Yiu K.H., Lau K.K., Zhao C.T., Chan Y.H., Chen Y., Zhen Z., Wong A., Lau C.P., Tse H.F. (2014). Predictive value of high-sensitivity Tn-I for future adverse cardiovascular outcome in stable patients with type 2 diabetes mellitus. Cardiovasc. Diabetol..

[163] Zhang J., Li X., Zhang S., Wang Z., Tian R., Xu F., Chen Y., Li C. (2024). Distribution and prognostic value of high-sensitivity cardiac Tn T and I across glycemic status: A population-based study. Cardiovasc. Diabetol..

[164] Osredkar J., Bajrić A., Možina H., Lipar L., Jerin A. (2024). Cardiac Tns I and T as Biomarkers of Cardiomyocyte Injury—Advantages and Disadvantages of Each. Appl. Sci..

[165] Airaksinen J.K.E., Tuominen T., Paana T., Hellman T., Vasankari T., Salonen S., Junes H., Linko-Parvinen A., Pallari H.M., Strandberg M. (2024). Novel Tn fragmentation assay to discriminate between Takotsubo syndrome and acute myocardial infarction. Eur. Heart J. Acute Cardiovasc. Care.

[166] Couch L.S., Garrard J.W., Henry J.A., Kotronias R.A., Alaour B., De Maria G.L., Channon K.M., Banning A.P., Lyon A.R., Marber M. (2024). Comparison of Tn and natriuretic peptides in Takotsubo syndrome and acute coronary syndrome: A meta-analysis. Open Heart.

[167] Airaksinen K.E.J., Aalto R., Hellman T., Vasankari T., Lahtinen A., Wittfooth S. (2022). Novel Tn Fragmentation Assay to Discriminate Between Tn Elevations in Acute Myocardial Infarction and End-Stage Renal Disease. Circulation.

[168] Brattström L., Wilcken D.E. (2000). Homocysteine and cardiovascular disease: Cause or effect?. Am. J. Clin. Nutr..

[169] Refsum H., Nurk E., Smith A.D., Ueland P.M., Gjesdal C.G., Bjelland I., Tverdal A., Tell G.S., Nygård O., Vollset S.E. (2006). The Hordaland Homocysteine Study: A community-based study of homocysteine, its determinants, and associations with disease. J. Nutr..

[170] Jin N., Huang L., Hong J., Zhao X., Chen Y., Hu J., Cong X., Xie Y., Pu J. (2021). Elevated homocysteine levels in patients with heart failure: A systematic review and meta-analysis. Medicine.

[171] Djuric D., Jakovljevic V., Zivkovic V., Srejovic I. (2018). Homocysteine and homocysteine-related compounds: An overview of the roles in the pathology of the cardiovascular and nervous systems. Can. J. Physiol. Pharmacol..

[172] de Lemos J.A., Ayers C.R., Levine B.D., deFilippi C.R., Wang T.J., Hundley W.G., Berry J.D., Seliger S.L., McGuire D.K., Ouyang P. (2017). Multimodality Strategy for Cardiovascular Risk Assessment: Performance in 2 Population-Based Cohorts. Circulation.

[173] Sickan J., Aw T.C., Du S.X., Li J., Janel H., Beshiri A. (2020). Cardiovascular disease risk assessment with high-sensitivity cardiac Tn I and other biomarkers: An observational cohort study in Johor, Malaysia. Malays. J. Public Health Med..

[174] Alam N., Khan H.I., Chowdhury A.W., Haque M.S., Ali M.S., Sabah K.M., Amin M.G. (2012). Elevated serum homocysteine level has a positive correlation with serum cardiac Tn I in patients with acute myocardial infarction. Bangladesh Med. Res. Counc. Bull..

[175] Kumar A., Sharma P., Kumar P., Kumar A. (2019). Homocysteine: A newer and novel independent risk factor and cardiac marker for acute MI. Asian J. Pharm. Clin. Res..

[176] Hanoon A.H., Jassim A.K., Hashim N.A. (2024). The effect of homocysteine and Tn levels on the development and diagnosis of cardiovascular diseases. Int. J. Cardiol. Sci..

[177] Al-Obaidi M.K., Stubbs P.J., Collinson P., Conroy R., Graham I., Noble M.I.M. (2000). Elevated homocysteine levels are associated with increased ischemic myocardial injury in acute coronary syndromes. J. Am. Coll. Cardiol..

[178] Ashraf M.U., Aslam M., Ajmal M.R., Habib A. (2014). Correlation of serum homocysteine with cardiac Tn-T in patients with acute myocardial infarction. Int. Arch. Integr. Med..

[179] Cao R., Bai Y., Xu R., Ye P. (2014). Homocysteine is associated with plasma high-sensitivity cardiac Tn T levels in a community-dwelling population. Clin. Interv. Aging.

[180] El-Amrousy D., Hassan S., Hodeib H. (2018). Prognostic value of homocysteine and highly sensitive cardiac Tn T in children with acute heart failure. J. Saudi Heart Assoc..

[181] Li W.J., Chen X.M., Nie X.Y., Zhang J., Cheng Y.J., Lin X.X., Wu S.H. (2015). Cardiac troponin and C-reactive protein for predicting all-cause and cardiovascular mortality in patients with chronic kidney disease: A meta-analysis. Clinics.

[182] Adeoye M., Hamdallah H., Adeoye A.M. (2025). Homocysteine levels and cardiovascular disease risk factors in chronic kidney disease (CKD), hypertensive and healthy Nigerian adults: A comparative retrospective study. BMJ Open.

[183] Aakre K.M., Saeed N., Wu A.H.B., Kavsak P.A. (2020). Analytical performance of cardiac troponin assays—Current status and future needs. Clin. Chem. Acta.

[184] Clerico A., Zaninotto M., Aimo A., Padoan A., Passino C., Fortunato A., Galli C., Plebani M. (2025). Advancements and challenges in high-sensitivity cardiac troponin assays: Diagnostic, pathophysiological, and clinical perspectives: On behalf of the Italian Study Group on Cardiac Biomarkers. Clin. Chem. Lab. Med. (CCLM).

[185] Koechlin L., Boeddinghaus J., Lopez-Ayala P., Reber C., Nestelberger T., Wildi K., Spagnuolo C.C., Strebel I., Glaeser J., Bima P. (2024). Clinical and Analytical Performance of a Novel Point-of-Care High-Sensitivity Cardiac Troponin I Assay. JACC.

[186] Nauck M., Bisse E., Nauck M., Wieland H. (2001). Pre-analytical Conditions Affecting the Determination of the Plasma Homocysteine Concentration. Clin. Chem. Lab. Med..

[187] Refsum H., Smith A.D., Ueland P.M., Nexo E., Clarke R., McPartlin J., Johnston C., Engbaek F., Schneede J., McPartlin C. (2004). Facts and Recommendations about Total Homocysteine Determinations: An Expert Opinion. Clin. Chem..

[188] McCarthy C.P., Raber I., Chapman A.R., Sandoval Y., Apple F.S., Mills N.L., Januzzi J.L. (2019). Myocardial Injury in the Era of High-Sensitivity Cardiac Tn Assays: A Practical Approach for Clinicians. JAMA Cardiol..

[189] Krychtiuk K.A., Newby L.K. (2024). High-sensitivity cardiac Tn assays: Ready for prime time!. Annu. Rev. Med..

[190] Narayanan M.A., Garcia S. (2019). Role of High-sensitivity Cardiac Tn in Acute Coronary Syndrome. US Cardiol. Rev..

[191] Yuan S., Mason A.M., Carter P., Burgess S., Larsson S.C. (2021). Homocysteine, B vitamins, and cardiovascular disease: A Mendelian randomization study. BMC Med..

[192] Clarke R., Halsey J., Lewington S., Lonn E., Armitage J., Manson J.E., Bønaa K.H., Spence J.D., Nygård O., Jamison R. (2010). Effects of Lowering Homocysteine Levels With B Vitamins on Cardiovascular Disease, Cancer, and Cause-Specific Mortality: Meta-analysis of 8 Randomized Trials Involving 37,485 Individuals. Arch. Intern. Med..

[193] Zhou C., Wu J., Fang S. (2013). Meta-analysis of B vitamin supplementation on plasma homocysteine, cardiovascular and all-cause mortality. Clin. Nutr..

[194] Zhang C., Wang Z.Y., Qin Y.Y., Yu F.F., Zhou Y.H. (2014). Association between B vitamins supplementation and risk of cardiovascular outcomes: A cumulative meta-analysis of randomized controlled trials. PLoS ONE.

[195] Januzzi J.L., Mahler S.A., Christenson R.H., Rymer J., Newby L.K., Body R., Apple F.S., Morrow D.A., Jaffe A.S. (2019). Recommendations for Institutions Transitioning to High-Sensitivity Tn Testing. J. Am. Coll. Cardiol..

[196] de Haan J. (2019). Preparing for High Sensitivity Tn Testing. Acutecaretesting.org. https://acutecaretesting.org/en/articles/preparing-for-high-sensitivity-troponin-testing.

[197] Rasmussen K., Møller J. (2000). Total homocysteine measurement in clinical practice. Ann. Clin. Biochem..

[198] Jacques P.F., Bostom A.G., Wilson P.W., Rich S., Rosenberg I.H., Selhub J. (2001). Determinants of plasma total homocysteine concentration in the Framingham Offspring cohort. Am. J. Clin. Nutr..

[199] Brustolin S., Giugliani R., Félix T.M. (2010). Genetics of homocysteine metabolism and associated disorders. Braz. J. Med. Biol. Res..

[200] Filipovic M.G., Luedi M.M. (2023). Cardiovascular Biomarkers: Current Status and Future Directions. Cells.

[201] Pervan P., Svaguša T., Prkačin I., Savuk A., Bakos M., Perkov S. (2017). Urine high sensitive Tn I measuring in patients with hypertension. Signa Vitae.

[202] Mirzaii-Dizgah I., Riahi E. (2013). Salivary high-sensitivity cardiac Tn T levels in patients with acute myocardial infarction. Oral Dis..

[203] Lazar D.R., Lazar F.L., Homorodean C., Cainap C., Focsan M., Cainap S., Olinic D.M. (2022). High-Sensitivity Tn: A Review on Characteristics, Assessment, and Clinical Implications. Dis. Markers.

[204] Kongintr U., Lertanantawong B., Promptmas C. (2023). A Label-Free Electrochemical Biosensor for Homocysteine Detection Using Molecularly Imprinted Polymer and Nanocomposite-Modified Electrodes. Polymers.

[205] Zaimbashi R., Tajik S., Beitollahi H., Torkzadeh-Mahani M. (2023). Fabrication of a Novel and Ultrasensitive Label-Free Electrochemical Aptasensor Based on Gold Nanostructure for Detection of Homocysteine. Biosensors.

[206] Piorino F., Johnson S., Styczynski M.P. (2023). A Cell-Free Biosensor for Assessment of Hyperhomocysteinemia. ACS Synth. Biol..

[207] Bularga A., Lee K.K., Stewart S., Ferry A.V., Chapman A.R., Marshall L., Strachan F.E., Mills N.L., Anand A. (2019). High-Sensitivity Tn and the Application of Risk Stratification Thresholds in Patients With Suspected Acute Coronary Syndrome. Circulation.

[208] Marston N.A., Bonaca M.P., Jarolim P., Goodrich E.L., Bhatt D.L., Steg P.G., Cohen M., Storey R.F., Johanson P., Wiviott S.D. (2020). Clinical Application of High-Sensitivity Tn Testing in the Atherosclerotic Cardiovascular Disease Framework of the Current Cholesterol Guidelines. JAMA Cardiol..

[209] Bhatia P.M., Daniels L.B. (2020). Highly Sensitive Cardiac Tns: The Evidence Behind Sex-Specific Cutoffs. J. Am. Heart Assoc..

[210] Neumann J.T., Twerenbold R., Ojeda F., Aldous S.J., Allen B.R., Apple F.S., Babel H., Christenson R.H., Cullen L., Di Carluccio E. (2025). ARTEMIS study group (2023). Personalized diagnosis in suspected myocardial infarction. Clin. Res. Cardiol..

[211] Toprak B., Solleder H., Di Carluccio E., Greenslade J.H., Parsonage W.A., Schulz K., Cullen L., Apple F.S., Ziegler A., Blankenberg S. (2024). Diagnostic accuracy of a machine learning algorithm using point-of-care high-sensitivity cardiac troponin I for rapid rule-out of myocardial infarction: A retrospective study. Lancet Digit. Health.

[212] Si T., Zhang W., Fu X., Wang Y., Liu D., Wu Q. (2022). Reference intervals of homocysteine in apparently healthy Chinese Han ethnic adults. J. Lab. Med..

[213] Ma C., Li L., Wang X., Hou L., Xia L., Yin Y., Cheng X., Qiu L. (2022). Establishment of Reference Interval and Aging Model of Homocysteine Using Real-World Data. Front. Cardiovasc. Med..

[214] Cacciapuoti F. (2011). Hyper-homocysteinemia: A novel risk factor or a powerful marker for cardiovascular diseases? Pathogenetic and therapeutical uncertainties. J. Thromb. Thrombolysis.

[215] González-Lamuño D., Arrieta-Blanco F.J., Fuentes E.D., Forga-Visa M.T., Morales-Conejo M., Peña-Quintana L., Vitoria-Miñana I. (2024). Hyperhomocysteinemia in Adult Patients: A Treatable Metabolic Condition. Nutrients.

[216] Koklesova L., Mazurakova A., Samec M., Biringer K., Samuel S.M., Büsselberg D., Kubatka P., Golubnitschaja O. (2021). Homocysteine metabolism as the target for predictive medical approach, disease prevention, prognosis, and treatments tailored to the person. EPMA J..

[217] Nguyen K., Fan W., Bertoni A., Budoff M.J., Defilippi C., Lombardo D., Maisel A., Szklo M., Wong N.D. (2020). N-terminal Pro B-type Natriuretic Peptide and High-sensitivity Cardiac Troponin as Markers for Heart Failure and Cardiovascular Disease Risks According to Glucose Status (from the Multi-Ethnic Study of Atherosclerosis [MESA]). Am. J. Cardiol..

[218] Dupuy A.M., Curinier C., Kuster N., Huet F., Leclercq F., Davy J.M., Cristol J.P., Roubille F. (2016). Multi-Marker Strategy in Heart Failure: Combination of ST2 and CRP Predicts Poor Outcome. PLoS ONE.

[219] Cho D.-Y., Kim K.-N., Kim K.-M., Lee D.-J., Kim B.-T. (2012). Combination of high-sensitivity C-reactive protein and homocysteine may predict an increased risk of coronary artery disease in Korean population. Chin. Med. J..

[220] Wang K., Wang Y., Chu C., Hu J., Zheng W., Yan Y., Ma Q., Gao K., Yuan Y., Mu J. (2019). Joint Association of Serum Homocysteine and High-Sensitivity C-Reactive Protein with Arterial Stiffness in Chinese Population: A 12-Year Longitudinal Study. Cardiology.

[221] Djuric D.M., Todorovic D., Bajic Z., Krneta S.M., Sobot T., Djuric D.M., Agrawal D.K. (2024). Is Homocysteine a Biomarker of Environmental Health Risk and Epigenetic-DNA Methylation: Links to Cardiovascular Pathogenesis and B Vitamins. Environmental Factors in the Pathogenesis of Cardiovascular Diseases. Advances in Biochemistry in Health and Disease.

